# Exploratory analysis of nutrient composition of adult and senior dog diets

**DOI:** 10.3389/fvets.2025.1717409

**Published:** 2025-12-18

**Authors:** Kyle German, Cynthia Melgoza, Camille Torres-Henderson

**Affiliations:** 1 Department of Clinical Sciences, Colorado State University, Fort Collins, CO, United States; 2 Rising Sun Animal Care, Denver, CO, United States

**Keywords:** canine nutrition, senior dogs, pet food formulation, nutrient requirements, AAFCO, nutrient analysis

## Abstract

**Introduction:**

Senior dog foods are often marketed as distinct from adult formulations, yet no specific nutrient profiles exist for this life stage. This study evaluated nutrient composition of over-the-counter adult and senior canine diets in relation to Association of American Feed Control Officials (AAFCO) adult maintenance requirements.

**Methods:**

Sixty-one dry and canned diets were purchased from retail stores and complete proximate analysis with minerals was performed. The diets selected consisted of 25 brands (companies), including 30 diets marketed for adults (21 dry, 8 canned, and 1 freeze-dried) and 31 diets marketed for seniors (25 dry, 6 canned). Diets were randomly selected using a random number generator from the complete inventory of dog foods available at two pet food stores in Fort Collins, Colorado (one national retailer and one locally owned store). Nutrients were expressed per 1,000 kcal metabolizable energy, and descriptive statistics were generated. Adult vs. senior, dry vs. canned, and combined categories were compared using parametric or non-parametric tests depending on distribution. Within each company, paired adult and senior products were also evaluated for percent change in nutrient content.

**Results:**

Eighteen percent (18%; 11/61) of products did not meet at least one requirement for AAFCO adult maintenance, and differences between adult and senior diets were inconsistent. Fiber, fat, and energy density showed the greatest variability, especially among canned diets.

**Discussion:**

As a descriptive exploratory analysis, no power calculation or adjustment for multiple comparisons was performed, and results should be interpreted as hypothesis generating. Overall, senior dog diets demonstrated wide variability and did not consistently differ from adult products, underscoring the challenge with not having defined nutrient profiles for this life stage, and the need for further research linking diet composition to clinical outcomes in aging dogs.

## 1 Introduction

The term “senior” is used to describe the older, aging pet; however, there isn't a specific age threshold for senior status in dogs due to the variability in aging across breeds and sizes of dogs (1). Providing care for pets as they age is a common concern for owners, and veterinarians are often asked when it is appropriate to transition their dog to a senior diet; however, standardized nutrient profiles for senior dogs do not exist and as a result the nutrient composition for a senior diet is determined by the individual manufacturer. One survey reported approximately 40% of owners with older dogs feed a diet marketed for senior dogs, with 33% of those doing so on advice from their veterinarian (2). Additionally, 85% of owners believe senior dogs have distinct nutritional needs that differ from younger adult dogs (2). With dogs 7 years and older comprising approximately half of the canine population, there is strong interest in nutrition for the senior life stage, although evidence based nutrient requirement guidelines remain limited (1, 3).

Reaching a consensus on what constitutes an ideal senior dog diet is challenging because senior status does not have a universally accepted definition, older dogs may have multiple comorbidities, and dogs age at different rates, with large breed dogs typically aging faster than small breed dogs. One commonly referenced definition comes from the 2019 AAHA Canine Life Stage Guidelines which defines senior as “the last 25% of estimated lifespan through end of life” (4). In contrast, The Dog Aging Project defines seniors as 11 or older for statistical analysis purposes (3). Whereas others suggest a species wide definition that classifies senior as all dogs between 7 and 11 years of age regardless of breed or size (5). This means veterinarians should work closely with owners to treat each aging dog as a unique individual and determine when nutritional changes might be needed.

Neither the Association of American Feed Control Officials (AAFCO), nor the National Research Council (NRC) have established a definition for senior life stage and as such, specific nutrient profiles for senior pets do not exist (6, 7). As a result, dog food marketed for seniors is formulated to meet the nutrient requirements for adult maintenance, or all life stages. This leaves decisions regarding nutrient profile of diets marketed for senior dogs to the discretion of the pet food manufacturer. Each manufacturer must determine what they believe a senior diet should be, which leads to wide nutrient variability and little consensus amongst diets marketed for senior dogs. While senior diets are formulated to be complete and balanced for adult dogs, pet owners or veterinarians may assume these diets are specifically designed to address age-related changes or medical conditions that are common in senior pets. Using a senior diet with this intention has the risk that the nutrient composition may not match the individual needs of a dog with age related or specific health concerns.

The European Pet Food Industry Federation (FEDIAF) released general guidelines in 2017 that encouraging manufacturers to consider lowering energy density and increasing dietary protein, fiber and omega-3 fatty acids in senior dog diets; however, no specific requirements were implemented due to limited data (8, 9). In many dogs energy requirements can decrease with age, which can predispose dogs to obesity (10, 11). Because maintaining a healthy body condition is associated with decreased morbidity and mortality, diets lower in energy density may benefit many senior dogs (12). However, not all aging dogs experience reduced energy needs due to variability in body composition, activity level, and health status. Some senior dogs, particularly those with chronic disease, experience a decreased appetite or those approaching the end of their lifespan may struggle to maintain weight, requiring an energy dense diet to support a healthy body condition (8).

FEDIAF suggests increasing dietary protein, when not medically contraindicated, to support lean body mass (8). Older dogs have increased protein turnover, necessitating the need for more protein (10, 11, 13, 14). Because caloric restriction to manage excess body weight reduces the intake of all nutrients, ensuring adequate dietary protein becomes especially important. If calories are restricted without increasing protein concentration, protein intake may not meet minimum requirements. Higher dietary crude fiber is also recommended to promote proper gastrointestinal motility and to decrease the energy density (8).

Previous work evaluating the nutrient profile of cat diets found that 12% of the diets evaluated did not meet their nutritional adequacy statement (15). The group also found that diets marketed for seniors only had minor differences than those for adults (15–17). Additional studies that compared nutrient analysis of over the counter dog foods against AAFCO maintenance requirements found that 13% of diets failed to meet minimum requirements (18). These findings highlight the need to evaluate how senior dog diets compare with adult diets and whether they meet established nutritional guidelines. To provide broader context for commercial diet characteristics, market segment was also included as a descriptive variable in this study. To our knowledge, no peer-reviewed study has systematically compared the nutrient profiles of commercially available adult and senior canine diets across life-stage, diet-format, and market-segment categories. The objective of this study was to analyze nutrient profiles of over-the-counter dog diets and determine whether differences exist between senior and adult diets, between canned and dry formulations, across market segments, and whether these diets meet AAFCO adult maintenance requirements. Because no prior work has addressed these comparisons, this study was designed as a descriptive, exploratory analysis. Based on previous literature, we anticipated that nutrient profiles of senior dog diets would not differ significantly from adult diets, although modest reductions in energy density, crude fat, crude protein, and phosphorus might be observed, with wide considerable variability across formulations.

## 2 Materials and methods

For this descriptive study, a list of 1,118 canine diets representing 53 brands available for purchase in May 2023 was compiled from two pet food stores in Fort Collins, Colorado, including one national retail chain and one independent local retailer. These retail sources were selected to capture a range of market segments, including large-scale national brands and smaller specialty products. Diets were purchased in May 2023 from one of these two retail sources that represented different distribution models: a national pet specialty retailer and a local independent pet food store. From this list, 61 canine diets were randomly selected using a randomized number generator, representing 25 brands. Of these, 30 diets were marketed for adult dogs (21 dry, 8 canned, and 1 freeze-dried), and 31 diets were marketed for senior dogs (25 dry and 6 canned). A diet was determined to be in the senior diet group based on marketing by the pet food manufacturer that included labels such as: 7+, senior, mature adult, etc. All 61 diets were formulated to meet the AAFCO nutrient levels for adult maintenance based on the nutritional adequacy statement on the label.

All diets used in the study were purchased and analyzed in May of 2023. A portion of each diet (250 g) was sent to Midwest Laboratories^a^ for complete proximate analysis with minerals. Samples were labeled with a randomly assigned number to blind product identity. The nutrient analysis for all diets were compared to AAFCO maintenance requirements based on calorie basis which allows more accurate comparisons between diets with varying energy densities. Energy density was calculated and compared on a dry matter basis (DM) across groups.

All diets were homogenized before analysis. Moisture was measured using AOAC 930.15, a loss on drying technique where the sample stayed in a 135 °C convection oven for 2 h. Crude protein was measured by Dumas combustion method, AOAC 990.03, which measured nitrogen levels. A multiplication factor of 6.25 was used to calculate crude protein from the measured nitrogen content. Crude fiber was measured using the Ankom filter bag technique, method AOCS Ba 6a-05. Crude fat was measured using acid hydrolysis, AOAC method 954.02, in which the sample was treated with ethanol and hydrochloric acid to help release fat followed by treatments of ethyl ether and petroleum ether to extract the fat. Mineral concentrations were measured using ICP-OES (inductively coupled plasma–optical emission spectroscopy), AOAC method 985.01. Samples were prepared using a wet ash procedure that requires acids and heat before the samples were analyzed with the ICP using a Thermo ICAP 6500 machine. Ash was measured using AOAC 942.05 where the sample was placed in a 600 °C muffle furnace. Carbohydrate content was estimated by difference, subtracting the percentages of protein, fat, crude fiber, moisture, and ash from 100%, which represents the nitrogen-free extract (an estimate of the total non-fiber carbohydrate). Energy density was calculated using modified Atwater factors: 3.5 kcal/g for protein, 8.5 kcal/g for fat, and 3.5 kcal/g for carbohydrate (6).

Market classification was used to describe differences in manufacturer scale and retail distribution characteristics (Table 1). These criteria were selected because annual revenue is a widely used indicator of company size, while ownership and retail distribution provide consistent secondary information when revenue data are unavailable. Company revenue data were obtained from publicly available industry reports and financial statements, with all values converted to U.S. dollars in billions for consistency (19). When a pet food brand was owned by a larger parent company, the parent company's reported annual revenue from its pet food division was used to determine categorization. This allowed for consistent grouping of brands under the same corporate entity, reflecting their combined market influence and distribution reach. Brands were classified into three market categories based on estimated annual revenue: mass-market (>$4 billion), mid-market ($0.5–3.9 billion or not listed but with broad retail availability), and selective-market (>$0.5 billion or limited distribution). For brands without publicly reported revenue, classification was based on consistent secondary criteria incorporating company ownership and predominant retail distribution channels. In these cases, products available nationally through major retailers (e.g., PetSmart, Petco, Chewy) were classified as mid-market, whereas those with limited distribution through independent retailers or direct-to-consumer sales were classified as selective-market. Because brands were represented by varying numbers of diets in the sample, market category distribution was summarized at both the brand and diet levels.

**Table 1:** Rubric for market segment classification of pet food manufacturers.

**Criterion**	**Mass-market**	**Mid-market**	**Selective-market**
Primary Classification Basis	Annual revenue used as primary determinant	Annual revenue used as primary determinant	Annual revenue used as primary determinant
Annual Revenue (billion USD)	≥4.0	0.5–3.9 or not publicly reported but with broad national retail availability	< 0.5 or not publicly reported and limited retail distribution
Manufacturer Ownership	Often subsidiaries of large multinational corporations	May be privately owned or part of a regional or national company; may belong to smaller corporate groups	Often independently owned or small companies; may include privately or family-owned businesses
Primary Retail Distribution Channels	Widely available through mass retail and major e-commerce platforms	National distribution through pet specialty retailers and major online platforms (e.g., Chewy, PetSmart, Petco)	Distributed mainly through independent retailers, small specialty outlets, or direct-to-consumer/online sales

Rubric used to classify pet food manufacturers into market segments. Classification was based primarily on reported annual revenue (in billions of USD), with manufacturer ownership and retail distribution channels used as secondary criteria to guide categorization when revenue data were unavailable.

## 3 Statistical analysis

Because AAFCO does not specify nutrient requirements for senior diets, all nutrient concentrations were compared to AAFCO requirements for adult maintenance. AAFCO does not have requirements for energy density, crude fiber, or carbohydrates, so comparisons were made between the groups. Vitamins, choline, chloride, iodine, selenium, individual fatty acids, and amino acids were not analyzed and therefore were not included in the AAFCO comparison. For each AAFCO nutrient requirement, descriptive data was recorded for each diet as a binary variable, with each nutrient treated as an individual data point.

Nutrient concentrations of all diets were compared to AAFCO adult maintenance requirements on an energy basis (g or mg/1,000 kcal ME), with energy density expressed on a kcal/kg dry matter (DM) basis. Comparisons were made at several levels, including: all adult diets (dry + canned) vs. all senior diets (dry + canned), all dry diets vs. all canned diets, adult dry vs. senior dry diets, and adult canned vs. senior canned diets. In addition, for brands with both adult and senior dry diets, percent change in nutrients was calculated to assess trend within the company. All nutrient data was summarized as mean +/- standard deviation (SD), median, and range (minimum–maximum) and expressed in nutrient per 1,000 kcal basis. Interquartile range was determined to assess the middle 50% of the data. The normality was assessed using the Shapiro–Wilk test for each nutrient within adult dry, senior dry, adult canned, and senior canned, as well as the combined groups used for analysis comparing all adult, all senior, all dry, all canned. Because many nutrient distributions demonstrated non-normality, often due to right-skew from high outliers, non-parametric tests were applied. Both parametric and non-parametric analyses were performed. Welch's *t*-test (two-sided, unequal variance assumed) was used to compare nutrient densities between groups when the data was approximately normal. Mann–Whitney *U* tests were used as a non-parametric alternative to address nutrients not normally distributed. For those skewed nutrients, results were interpreted with greater emphasis on median values and non-parametric tests. Statistical significance was set at *p* < 0.05 for both the Welch's and Mann–Whitney *U* tests.

One freeze-dried adult diet was excluded from the statistical analysis comparing diet types due to its classification as a significant outlier in several categories, including energy density, carbohydrate, and fat content. As the only freeze-dried product in the dataset, its removal allowed for a more accurate comparison among dry and adult diet categories. However, the diet was kept in for the AAFCO comparison because all diets with an adult-maintenance AAFCO nutritional adequacy statement, are expected to meet the same requirements regardless of product form. Including this diet in the AAFCO evaluation therefore allowed for a complete assessment of whether every diet in the dataset met the established adult-maintenance standards.

Seven companies with both dry adult and dry senior diets were compared to one another with paired adult–senior formulations to evaluate for nutrient trends. The only analysis calculated from this information was the percent change in nutrients between adult dry diets compared to senior dry diets. Line graphs were generated to illustrate these trends, with each company represented by a consistent color. This allowed for visual evaluation of patterns within each company, independent of data from other companies. When a company had more than one diet within the adult or senior category, average nutrient values were calculated and used to represent that category for comparison purposes. Only one company in the sample group had both adult and senior canned diets, which did not provide sufficient data for statistical comparison.

The outcome data on the nutrients were described using mean and standard deviation. Normality was evaluated using Shapiro-Wilk statistics. If normality was met, the data between dry and canned were compared using Welch's *t*-test. If normality was not met, Mann–Whitney *U* testing was used to evaluate the difference between two diet groups. The data was analyzed using GraphPad Prism 10.6.1 statistical program. A *p*-value of 0.05 was used to determine statistical significance. Adult vs. senior diets were described and represented graphically.

Descriptive statistics were used to summarize market category distributions across all diets. Categorical variables (e.g., market classification, and life stage designation) were reported as frequencies and percentages. Because the number of diets included per brand varied within the sample, market-related results were examined at both the brand level (25 brands) and the diet level (61 diets) to distinguish differences attributable to manufacturers from variation among individual formulations.

## 4 Results

### 4.1 Market classification

A total of 61 canine diets were analyzed, representing 30 adult and 31 senior formulations across 25 brands. At the brand level, 6 of the 25 brands (24%) were classified as mass-market, 8 (32%) as mid-market, and 11 (44%) as selective market. At the diet level, 23 of the 61 diets (38%) were categorized as mass-market, 18 (30%) as mid-market, and 20 (33%) as selective-market.

### 4.2 Comparison with AAFCO requirements for adult maintenance

The nutrient analysis of all diets was compared to AAFCO requirements for canine adult maintenance. Eleven of 61 diets (18%) did not meet AAFCO requirements for at least one nutrient; of these, five diets had multiple nutrients outside the requirements (2 from mid-market group and 3 from selective market group). In total, there were 17 instances of nutrients not meeting AAFCO requirements, all involving calcium, phosphorus, potassium, or the calcium-to-phosphorus (Ca:P) ratio. Five diets (four adult, one senior) did not meet the minimum potassium requirement of 1.5 g/1,000 kcal. with potassium content ranging from 1.0 to 1.3 g/1,000 kcal (67%−87% of AAFCO minimum). Six diets (three adult, three senior) exceeded the maximum calcium level of 6.25 g/1,000 kcal, and four diets (1 adult, 3 senior) had phosphorus levels above 4.0 g/1,000 kcal. Calcium concentrations in diets exceeding the maximum ranged from 6.4 to 8.9 g/1,000 kcal (103%−142% above AAFCO maximum) including two diets from the same company that contained more than 7.8 g/1,000 kcal. Phosphorus concentrations above the maximum ranged from 4.1 to 4.4 g/1,000 kcal (103%−110% above AAFCO maximum). Two adult diets had a calcium-to-phosphorus ratio >2:1 (2.2:1 and 2.3:1), no diets had a ratio below 1. One diet had three nutrients that did not meet AAFCO adult maintenance requirements, including insufficient potassium, excessive calcium, and an elevated calcium-to-phosphorus (Ca:P) ratio. Table 2 summarizes the comparison of nutrient analysis to AAFCO adult maintenance along with median and range values for each of the four diet groups.

**Table 2:** Nutrient concentrations of adult and senior dog diets (dry and canned) compared to 621 AAFCO adult maintenance requirements.

**Nutrient**	**AAFCO requirement (units/1000 kcal)**	**Diets below AAFCO (n)**	**Diets above AAFCO (n)**	**Dry adult median (range)**	**Dry senior median (range)**	**Canned adult median (range)**	**Canned senior median (range)**
Crude protein (g/1,000 kcal)	≥ 45	0	N/A^*^	76 (63–101)	75 (52–116)	89 (62–128)	89 (69–111)
Crude fat (g/1,000 kcal)	≥ 13.8	0	N/A^*^	41 (33–52)	37 (30–49)	61 (33–83)	60 (37–78)
Calcium (g/1,000 kcal)	1.25–6.25	0	6	4.1 (2.2–8.9)	4.0 (1.7–7.8)	4.2 (2.5–6.8)	4.3 (2.6–5.9)
Phosphorus (g/1,000 kcal)	1.0–4.0	0	4	2.8 (1.5–4.3)	3.0 (1.7–4.4)	2.7 (1.8–3.2)	3.1 (2.0–3.6)
Ca:P ratio	1:1–2:1	0	2	1.4 (1.1–2.3)	1.4 (1–1.8)	1.6 (1.1–2.2)	1.3 (1.1–1.8)
Potassium (g/1,000 kcal)	≥ 1.5	5	N/A^*^	2.2 (1.4–4.3)	2.2 (1.3– 3.4)	2.4 (1.0–4.3)	2.7 (2.3–3.2)
Sodium (g/1,000 kcal)	≥ 0.2	0	N/A^*^	0.9 0.5–1.4)	0.9 (0.3–1.7)	1.2 (0.3–1.4)	1.4 (0.7–2.7)
Magnesium (g/1,000 kcal)	≥ 0.15	0	N/A^*^	0.4 (0.3–0.6)	0.4 (0.3–0.8)	0.2 (0.2–0.3)	0.2 (0.2–0.4)
Iron (mg/1,000 kcal)	≥ 10	0	N/A^*^	57 (34–134)	56 (29–109)	70 (37–94)	70 (42–105)
Copper (mg/1,000 kcal)	≥ 1.83	0	N/A^*^	4.5 (3.4–9.1)	4.6 (2.5–8.1)	6.2 (2.7–7.7)	6.3 (4.8–13.1)
Manganese (mg/1,000 kcal)	≥ 1.25	0	N/A^*^	9.1 (6.1–27.9)	10.4 (3.3–30.8)	6.6 (2.9–7.9)	6.3 (3.6–20.1)
Zinc (mg/1,000 kcal)	≥ 20	0	N/A^*^	52 (31–114)	56 (30–95)	64 (36–92)	54 (44–70)

Nutrient concentrations of adult and senior dog diets compared to AAFCO adult maintenance requirements (per 1000 kcal). Values represent observed median and ranges for each category. Numbers in “below minimum” and “above maximum” columns indicate the number of diets outside AAFCO requirements. Ca:P ratio expressed as calcium-to-phosphorus.

^*^N/A indicates no maximum requirement established by AAFCO.

Analysis of the market categories revealed additional patterns in AAFCO compliance across brands. Across the 25 brands (6 mass-market, 8 mid-market, and 11 selective-market), 9 brands (36%) had at least one non-compliant diet. None of the mass-market brands had diets that failed to meet AAFCO requirements. In the mid-market category, 3 of 8 brands (38%) had at least one non-compliant diet, whereas in the selective-market category, 6 of 11 brands (55%) were non-compliant. Two selective-market brands each had more than one non-compliant diet. One of these brands had calcium levels exceeding the maximum allowable limit in both of its evaluated diets (one adult and one senior), which were the only formulations from that company included in the dataset.

At the diet level, the 61 diets in the dataset included 23 mass-market, 18 mid-market, and 20 selective-market diets. All 23 mass-market diets met AAFCO nutrient standards for adult maintenance. In contrast, 3 of the 18 mid-market diets (17%) and 8 of the 20 selective-market diets (40%) did not meet one or more AAFCO nutrient standards, either by falling below required minimums or exceeding maximum allowable limits.

### 4.3 Energy density

Energy concentrations ranged from 3,717 to 5,595 kcal/kg DM in adult diets and 3,572–5,038 kcal/kg DM in senior diets. Median (IQR) values were 3,993 (3,893–4,179) for adult and 3,844 (3,791–3,990) for seniors (kcal/kg DM). Differences in energy density between adult and senior diets were statistically significant, with adult diets having a higher energy density (*p* = 0.01). Within the senior group, canned diets ranged from 3,815to 5,038 kcal/kg DM and dry diets from 3,572 to 4,152 kcal/kg DM, with canned diets having significantly higher energy density (*p* = 0.04). When compared within diet types, adult dry diets had significantly higher energy density than senior dry diets (mean 3,962 kcal/kg DM vs. 3,843 kcal/kg DM; *p* = 0.001) but there were no significant differences between adult and senior canned diets (mean 4,525 kcal/kg vs. 4,475 kcal/kg DM; *p* = 0.87). Finally, across all diets, canned diets had significantly higher energy density than dry diets (4,503 kcal/kg vs. 3,897 kcal/kg DM; *p* = 0.001). Summary statistics for energy density are presented in Table 3. A boxplot graph comparing kcal/kg DM of adult dry, senior dry, adult canned and senior canned diets can be found in Supplementary Figure 2.

**Table 3:** Mean and median caloric density (kcal/kg dry matter) plus range for adult and senior diets across dry and canned formulations.

**Diet type**	**Mean kcal/kg DM**	**Median kcal/kg DM**	**kcal/kg Range DM**
All adult	4,117	3,993	3,717–5,595
All senior	3,965	3,844	3,572–5,038
Adult dry	3,962	3,943	3,717–4,249
Adult canned	4,525	4,512	3,812–5,595
Senior dry	3,843	3,807	3,572–4,152
Senior canned	4,475	4,594	3,815–5,038
All dry	3,897	3,887	3,572–4,249
All canned	4,503	4,512	3,812–5,595

Mean caloric density (kcal/kg dry matter) and range for adult and senior canine diets across dry and canned formulations.

### 4.4 Proximate analysis

Crude fiber concentration tended to be higher in senior diets compared to adult diets, 8.5 (6.6–11.2) vs. 7.2 (5.6–8.9), but did not reach statistical significance (*p* = 0.08). Ash concentrations did not differ significantly between adult and senior diets (*p* = 0.79). Crude protein concentrations for adult diets 76 g/1,000 kcal (62–128 g/1,000 kcal) compared to senior diets 76 g/1,000 kcal (52–116 g/1,000 kcal) did not differ significantly (*p* = 0.88); however, crude fat concentrations were significantly higher in adult diets 44 g/1,000 kcal (33–83 g/1,000 kcal) compared to senior diets 37 g/1,000 kcal (30–78 g/1,000 kcal; *p* = 0.019). When comparing senior diets, canned formulations 60 g/1,000 kcal (37–78 g/1,000 kcal) had significantly more fat than dry diets 37 g/1,000 kcal (30–49 g/1,000 kcal; *p* = 0.012). Differences in carbohydrates between adult and senior diets were not significant but trended toward senior diets having more carbohydrates (*p* = 0.073). Within the senior group, dry diets had significantly more carbohydrates than canned diets (*p* < 0.001). Within the adult group, canned diets had significantly more fat 61 g/1,000 kcal (33–83 g/1,000 kcal) compared to dry diets ~41 g/1,000 kcal (33–52 g/1,000 kcal) (*p* = 0.03) while dry diets had significantly higher crude fiber 8.1 g/1,000 kcal (4.5–15.8 g/1,000 kcal) and carbohydrates 111 g/1,000 kcal (64–143 g/1,000 kcal) compared to canned diets (*p* = 0.02, *p* = 0.003 respectively). Summary statistics for proximate analysis of each category of diets can be found in Supplementary Table 4 (means), and Supplementary Table 5 (ranges). Boxplot graphs comparing proximate analysis results across diet categories can be found in Supplementary Figure 2.

### 4.5 Minerals

Phosphorus concentrations were not significantly different between adult and senior dog diets (*p* = 0.73). Calcium concentrations and Ca:P ratios did not differ significantly between adult and senior diets (*p* = 0.71 and *p* = 0.18, respectively). Likewise, no significant differences were observed between canned and dry senior diets (*p* = 0.84 and *p* = 0.86, respectively) or between canned and dry adult diets (*p* = 0.80 and *p* = 0.20, respectively). A subset of diets (7%; 4/61) exceeded the AAFCO maximum for phosphorus (≥4.0 g/1,000 kcal ME), and six diets exceeded the maximum for calcium (≥6.25 g/1,000 kcal ME); however, no diets had Ca:P ratios below the minimum requirement of 1.0. Magnesium concentrations did not differ significantly between adult and senior diets (*p* = 0.24); in the senior group, dry diets had significantly higher magnesium concentrations compared with canned diets (*p* = 0.002). This was also the case with the adult dry diets having more magnesium than canned diet (*p* < 0.001). Potassium and sodium concentrations did not differ significantly between adult and senior diets (*p* = 0.72 and *p* = 0.91, respectively) or between canned and dry adult diets (*p* = 0.37 and *p* = 0.32, respectively). Within senior diets there was no significant difference in sodium, but senior canned diets were significantly higher in potassium (*p* = 0.7 and *p* = 0.027, respectively). Five diets (8%) were below the AAFCO minimum for potassium.

Copper concentrations ranged from 2.7 to 9.1 mg/1,000 kcal in adult diets and 2.5–13.1 mg/1,000 kcal in senior diets, with median (IQR) values of 5.0 (4.2–6.5) and 4.8 (3.7–5.9), respectively. Differences between adult and senior diets were not significant (*p* = 0.51). Within senior diets, canned foods copper ranged from 4.8 to 13.1 mg/1,000 kcal and dry foods from 2.5 to 8.1 mg/1,000 kcal, with canned diets containing significantly more copper than dry (*p* = 0.02). Outliers at the upper end accounted for the higher values observed in both canned and dry diets. Iron, zinc, and manganese concentrations were not significantly different between adult and senior diets (*p* = 0.62, *p* = 0.99, and *p* = 0.21, respectively) or between canned and dry senior diets (*p* = 0.51, *p* = 0.46, and *p* = 0.14, respectively). Iron and zinc did not vary significantly between canned and dry adult diets (*p* = 0.24, *p* = 0.22, respectively) however dry adult diets had more manganese (*p* < 0.001). Summary statistics of minerals for each category of diets can be found in Supplementary Table 4 (means), and Supplementary Table 5 (ranges). Boxplot graphs comparing mineral levels of within diet categories can be found in Supplementary Figure 2.

### 4.6 Comparisons across diet categories

#### 4.6.1 All adult vs. all senior diets

When all adult diets (dry + canned) and all senior diets (dry + canned) were compared, energy density and crude fat were the only significant differences. Adult diets, 4,117 kcal/kg DM (3,717–5,595 kcal/kg DM), had higher energy density than senior diets, 3,965 kcal/kg DM (3,572–5,038 kcal/kg DM; *p* = 0.01). Senior diets contained significantly less fat (mean 42.2 g/1,000 kcal) than adult diets (mean 46.8 g/1,000 kcal, *p* = 0.019). Crude fiber and carbohydrate content trended toward being increased in senior diets, but the results didn't cross significance (*p* = 0.079 and *p* = 0.073, respectively). Mean crude protein was nearly identical for both groups (adult 80.9 g vs. senior 79.9 g). Each group had a wide, but similar, range for crude protein (62–128 g, 42–116 g/1,000 kcal), with the senior group shifted lower due to a few diets with lower protein content. Their medians and IQR spreads for protein were very similar, showing that most diets are comparable. The decrease in fat and increase in carbohydrates for senior diets likely reflects an attempt to decrease caloric density by pet food manufacturers. All minerals had nearly identical means, medians, and IQR spreads between the adult and senior groups. Nutrient means and ranges for all diet categories can be found in the Supplementary Tables 3, 4 respectively.

#### 4.6.2 Adult dry diets vs. senior dry diets

Adult dry diets had statistically higher concentrations of crude fat compared to senior dry diets (mean 42.4 g vs. 37.8 g per 1,000 kcal; *p* = 0.003). Senior dry diets numerically had higher carbohydrate and crude fiber concentration, but did not reach significance (*p* = 0.061 and *p* = 0.13, respectively). Senior diets demonstrated a higher IQR for crude fiber, reflecting both increased variability and a slight upward trend relative to adult diets. Crude protein concentrations were the same across both diet categories (both 78 g/1,000 kcal), but senior dry diets had a wider range in protein, with more diets on each extreme, suggesting some companies are increasing protein while others are lowering. All mineral concentrations were nearly identical between both groups.

#### 4.6.3 Adult canned diets vs. senior canned diets

No significant differences were observed in energy density or nutrient concentrations between adult and senior canned diets. Senior canned diets had wider nutrient ranges than adult canned diets, but median values were comparable.

#### 4.6.4 All dry diets vs. all canned diets

This comparison showed the greatest number of differences; however, due to the randomization focusing on adult or senior as parameters, there was an uneven representation of dry and canned diets. This imbalance does lead to limitations in statistical comparisons between dry and canned categories. Eight aspects of the diets (energy density, crude fat, crude fiber, carbohydrates, potassium, magnesium, manganese, and copper) showed significant differences between dry and canned diets (*p* < 0.05). Canned diets had higher average fat (40 g vs. 59 g/1,000 kcal; *p* < 0.001), potassium (2.3 g vs. 2.7 g/1,000 kcal; *p* = 0.025) and copper (4.9 mg vs. 6.3 mg/1,000 kcal; *p* = 0.023) than dry diets. While dry diets had higher crude fiber (8.9 g vs. 6.5 g/1,000 kcal; *p* = 0.014), carbohydrates (111 g vs. 53 g/1,000 kcal; *p* < 0.001), magnesium (0.4 g vs. 0.2 g/1,000 kcal; *p* < 0.001) and manganese (12.5 mg vs. 6.9 mg/1,000 kcal; *p* < 0.001) than canned diets. Canned diets tended to have more protein than dry diets (mean 78 g vs. 89 g/1,000 kcal), but the results were not significant (*p* = 0.063). There were no significant differences in ash, phosphorus, calcium, iron, and zinc. Nutrient means and ranges can be found in Supplementary Tables 3, 4.

#### 4.6.5 Adult dry vs. adult canned

Adult canned diets were significantly more energy dense than adult dry diets (4,512 kcal vs. 3,942 kcal/kg DM; *p* = 0.002). Adult canned diets had greater fat content and lower amounts of crude fiber and carbohydrates compared with adult dry diets (*p* = 0.03, *p* = 0.02, and *p* = 0.003, respectively). Adult canned diets also had a higher mean protein concentration (88 g vs. 78 g/1,000 kcal), although this difference was not statistically significant (*p* = 0.33). The wide range observed (62–128 g/1,000 kcal), along with the relatively small sample size of canned diets, may have contributed to the lack of significance (*p* = 0.33). Adult dry diets also had more magnesium and manganese than adult canned diets (*p* < 0.001 for both nutrients).

#### 4.6.6 Senior dry vs. senior canned diets

Senior canned diets had higher energy densities than senior dry diets (4,594 vs. 3,807 kcal/kg DM; *p* = 0.009). This difference is likely explained by the significantly greater fat content and lower carbohydrate content of the senior canned diets (*p* = 0.01 and *p* < 0.001, respectively). Canned senior diets also had higher median protein levels, 89 g vs. 74 g/1,000 kcal but it was not significant (*p* = 0.16). Senior canned diets contained higher potassium and copper levels than senior dry diets (*p* = 0.027, *p* = 0.02 respectively). Senior dry diets contained more magnesium than senior canned diets (*p* = 0.002).

### 4.7 Company differences

Company level comparisons were performed for the seven brands that had both adult dry and senior dry diets in our data set. Graphs for all nutrients are available in the Supplementary material, while those for calories, protein, and fiber are highlighted here, as these nutrients were considered nutrients of concern in FEDIAF's statement on senior dog nutrition (8).

**Figure 1:**
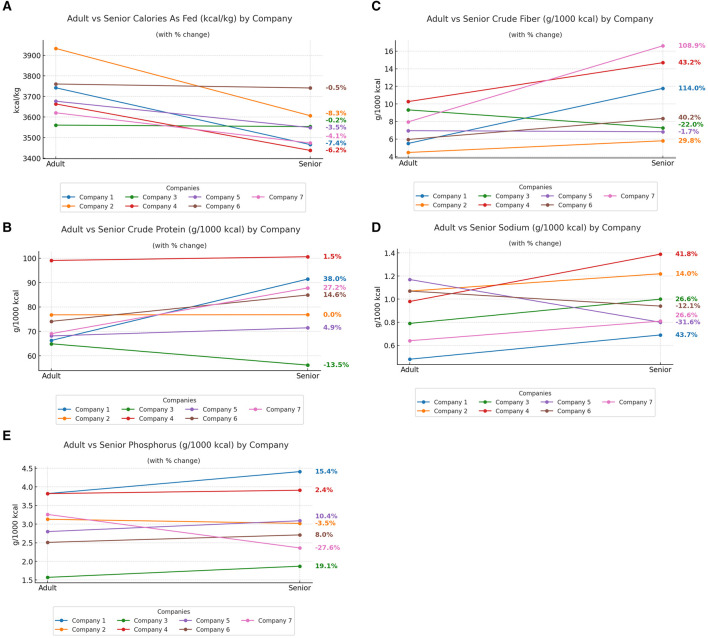
Comparison of nutrient content between adult and senior diets by company. Panels show **(A)** energy density, **(B)** crude protein, **(C) (C)** crude fiber, **(D)** sodium, and **(E)** phosphorus, expressed per 1,000 kcal. Each colored line represents an individual company's products, connecting adult and senior formulas for visual comparison. The lines do not represent time but serve as a visual aid to highlight the direction and magnitude of change within each company.

When evaluating trends within individual company there were several interesting findings. All companies reduced or essentially kept their energy density the same (< 1% change) as seen in Figure 1A. Six of the seven companies showed large reductions in fat which would match the lower energy density. Only one company's senior diet had lower protein than its adult. Five increased, while one remained unchanged (Figure 1B). Most companies increased the crude fiber concentration in their senior diets; however, one company reduced it by 21% and another by 2% (Figure 1C). Five of the seven companies increased sodium concentrations in their senior diets ranging from 14.4% to 43.7% (Figure 1D). Only two companies reduced phosphorus (27.6% and 3.5%), whereas the remaining companies increased phosphorus ranging from 2.4% and 19.7% (Figure 1E). Line graphs showing company changes for each nutrient analyzed can be found in Supplementary Figure 1.

## 5 Discussion

The results of this study demonstrate that OTC diets marketed for senior dogs show wide variability in nutrient composition and are similar to adult diets. Although the interquartile range (IQR) for several nutrients narrowed slightly in senior diets compared to adult diets, there was still substantial variation across all nutrients evaluated. This variability suggests that senior diets do not consistently address any single age-related issue. In contrast, therapeutic diets are formulated to target specific medical conditions. This emphasizes the importance of reviewing nutrient profiles and making specific diet recommendations for each individual patient rather than recommending all dogs over a certain age (e.g. ≥7 years) transition to a senior diet. When diet is being used to address age related changes or medical conditions, it is important to confirm that the new diet provides the nutrients in the desired range, rather than relying on a product labeled as a ‘senior diet'.

Metabolic changes associated with age can lead to a decrease in energy requirements, which can increase the risk for obesity (8, 10, 11, 13). The results indicate that some senior diets were lower in energy density and fat, which suggest some manufacturers have formulated senior diets to address this. It is important to consider that senior dogs have a wide variation in metabolic rate, activity level, and body composition. While many benefit from modest reductions in energy density, other dogs such as those with medical conditions, decreased appetite, or loss of lean body mass may require a diet with higher energy density to maintain optimal body weight and lean mass. Clinically, it is an important reminder that energy adjustments should be individualized rather than uniformly applied across this life stage. Although significant differences were observed in energy density (kcal/kg DM), the differences between adult and senior diets were not consistent, indicating that a diet labeled as “senior” cannot be assumed to provide fewer calories, emphasizing the importance of carefully screening and selecting diets for the intended use. Other aspects of senior diets that could suggest they are being formulated for weight control include the trends observed with higher concentrations of crude fiber and carbohydrates, which may help promote satiety.

The small differences observed between adult and senior diet groups could be explained by the lack of a standardized definition of a “senior” dog, and the lack of established specific nutritional requirements for this life stage. Without standardized nutrient profiles, manufacturers rely on internal formulation philosophies, leading to variation in nutrient content of senior diets and the potential for inconsistent alignment with age-related physiological needs. As a result, each company determines how diets marketed for senior pets are formulated. When comparing individual brands, we found that most companies formulated their senior diets differently from their adult diets, but the specific nutrient changes were company dependent and not uniform across brands. For energy density, crude protein, and crude fiber, there appeared to be general agreement among the seven brands within our dataset that offered both adult and senior diets, even though these differences were not statistically significant in the overall group comparisons. The lack of statistically significant differences that were observed between adult and senior diets at the group level may be attributed to the variability in manufacturer practices. Our analysis revealed that there were wide ranges in nutrients with similar medians and IQRs, suggesting that increases by some companies may have been offset by decreases from others. There were more significant differences between canned and dry diets than adult and senior diets. When a patient has a specific dietary requirement, it is important to evaluate for potential differences between canned and dry formulations that are marketed under the same product name to ensure that the selected formulation will address the patient's nutritional needs.

Based on these findings, it appears that while individual food companies may incorporate some of FEDIAF's recommendations for senior diets, the pet food industry in general does not consistently apply these principles. Which is not entirely unexpected with the dataset that was evaluated, as these were for diets sold in North America rather than the European Union. However, given the limited evidence available to establish optimal nutrient profiles for senior dogs, FEDIAF recommendations offer useful guidance for nutrients of concern in this population. FEDIAF recommends reducing energy density to account for the slower metabolic rates of aging dogs (8). Our results found that the energy density was lower in dry senior diets but not in canned senior diets. This could be related to the fact that canned senior diets were higher in crude fat which contributes to the energy density of the diet. Although FEDIAF and others have recommended increasing dietary protein concentrations in senior diets, our analysis did not identify a significant difference in crude protein between adult and senior formulations (8, 10, 11, 13, 14). When we evaluated what individual brands were changing between their adult and senior diets, we found there was not a consensus, even between the dietary factors that FEDIAF provides guidance on (energy density, crude protein, and crude fiber) (8). Although mean protein concentrations were essentially identical between adult and senior diets, the wide range observed in dry senior diets highlights inconsistency across manufacturers. Some companies appear to be lowering protein, while others are increasing it, resulting in no net difference at the group level. This variability supports the concern that the “senior” label does not reliably predict protein content and reinforces the need for veterinarians to assess diets individually. Crude fiber followed a similar pattern but had a less pronounced range than protein. Crude fiber did not have a significant difference between groups, but it is important to note that crude fiber only measures a portion of the insoluble fiber and does not include soluble fiber (20). If total dietary fiber was measured and compared, it is possible a difference between senior diets and adult diets would have been detected.

Even though crude fat was lower in senior diets compared to adult diets, 81% of senior diets would be classified as moderate fat (30–50 g/1,000 kcal) and 16% would be considered high fat (>50 g/1,000 kcal). Only one diet within this group would be considered low fat at 29.9 g/1,000 kcal (with low fat defined as < 30 g/1,000 kcal) (21). Therapeutic diets formulated to be low in fat provide 19.5–25 g/1,000 kcal (median of 21 g/1,000 kcal), which is lower than the median value observed in our dataset (37 g/1,000 kcal). For the nutritional management of conditions, such as hypertriglyceridemia, pancreatitis, or lymphangiectasia, over-the-counter diets are unlikely to be low enough in fat to effectively manage these cases.

A common misconception of senior diets is that they are lower in phosphorus to address renal changes associated with age. Our analysis found no significant difference in phosphorus concentration between adult and senior diets; however, the median value of phosphorus in senior diets was slightly higher than adult diets (3.0 vs. 2.8 g/1,000 kcal). While there is strong evidence to support phosphorus restriction once chronic kidney disease (CKD) is present, there is insufficient evidence to suggest early restriction will prevent or reduce the chances of a dog developing CKD (22, 23). The International Renal Interest Society (IRIS) does not recommend phosphorus restriction until a dog is in stage 2 CKD and has a serum phosphorus >4.6 mg/dL (24). Therapeutic diets formulated for dogs with CKD have a mean phosphorus of 0.76 g/1,000 kcal (range: 0.5–1.1 g/1,000 kcal). In contrast, the mean phosphorus concentration of the senior diets in our study was nearly four times higher. These findings suggest that OTC senior diets are not an appropriate substitute for therapeutic renal diets in the management of dogs with CKD.

Copper concentration in commercial dog food is controversial. Reports of an increased incidence of copper-associated hepatopathy have raised concerns about the potential role of dietary copper (25). This has led to calls for AAFCO to establish a maximum allowable limit for copper, similar to FEDIAF, which sets an upper limit of 2.8 mg/100 g DM (25, 26). FEDIAF does not provide the upper limit on a caloric basis but using their recommended conversions (2.5 x the mg/100 g DM) then this would equal an upper limit of 7 mg/1,000 kcal (26). Eleven of the diets in our group (18%) were over 7 mg/1,000 kcal. In addition, the copper concentration of all diets in our dataset ranged from 2.5 to 13.1 mg/1,000 kcal and the median copper concentration was about 5 mg/1,000 kcal. In contrast, therapeutic diets formulated for dogs with copper storage disease average 1.05 mg/1,000 kcal, with a narrow range of 0.9–1.2 mg/1,000 kcal. These findings highlight the marked differences between OTC and therapeutic diets, with some OTC formulations far exceeding the copper concentrations considered appropriate for managing copper-associated hepatopathy.

It is concerning that nearly one in five diets in our dataset failed to meet the nutrient requirements established by AAFCO for adult maintenance. From a clinical perspective, deviations from AAFCO nutrient minimums or excesses above maximums may adversely affect the dogs fed these diets. Deficiencies in essential nutrients can impair metabolic function, growth, or immune health, whereas excessive concentrations may contribute to toxicity or metabolic disturbances. We found that all diets within the mass-market group met AAFCO requirements, whereas the diets that did not meet AAFCO requirements were within the mid-market and selective-market groups. Because this study did not evaluate company-level practices and quality-control processes, no conclusions can be drawn about the reasons for these differences. These findings should be interpreted as descriptive associations rather than evidence of underlying causes. In general, manufacturers may differ in organizational structure, use of contract manufacturing, and quality-control practices, and such factors could theoretically influence nutrient consistency, but these aspects were not assessed in this dataset. Additional research would be needed to determine whether and how such factors relate to compliance with AAFCO requirements. When interpreting nutrient adequacy and variability across products, it is important to recognize that differences between products may reflect not only intentional formulation targets but also variation in manufacturer resources, production scale, and the reliability of ingredient databases; however, these factors were not directly evaluated in this study and should be considered hypothetical contributors. These observations highlight the importance of using accurate ingredient databases and analyzing nutrients of the finished product to confirm that diets meet established nutrient requirements. Our results found that multiple diets from the same company exceeded maximum calcium levels. This finding could indicate errors in the amount of mineral premix added or differences between measured ingredient calcium content and the values assumed in formulation software, although other contributing factors cannot be ruled out based on the available data. Most of the evidence regarding complications from excessive dietary calcium intake comes from studies in large and giant breed puppies, such as Great Danes. Large breed puppies fed free choice, high calcium diets, were at increased risk of developing skeletal abnormalities including osteochondrosis, decreased bone turnover, radius curvus, and stunted growth. Regardless of the calcium to phosphorus ratio, excess dietary calcium intake in large breed puppies has been shown to increase expression in all developmental orthopedic diseases including: hypertrophic osteodystrophy (HOD), osteochondrosis (OC), osteochondritis dissecans (OCD), retained cartilaginous core, panosteitis, hip dysplasia (HD), and elbow dysplasia (27). In general practice, it is common for clients with a newly acquired large-breed puppy to report that they were advised against feeding a puppy diet, out of concern for promoting excessively rapid growth. However, avoiding appropriately formulated large-breed puppy diets can increase the risk of developmental problems. Slater et al. found that feeding large-breed puppies diets with calcium concentrations above 3.6 g/1,000 kcal ME was associated with increased risk of osteochondritis (28). Thirty six of our 61 diets analyzed were above this threshold. AAFCO established calcium parameters for growth of large breed dogs to be 3.0 to 4.5 g/1,000 kcal, reflecting the narrow margin of safety required to minimize developmental orthopedic disease risk (7). Only 40% of the diets in our dataset fell within this range, with 15 diets falling below, and 21 diets exceeding this range. These findings demonstrate the importance of feeding diets with the proper life stage qualifiers, such as growth of large breed dogs. Feeding large breed puppies diets labeled for adult maintenance puts them at risk for nutrition related developmental orthopedic problems. Furthermore, these diets do not account for growth requirements of other essential nutrients, compounding the potential for poor outcomes in growing dogs.

When interpreting nutrient adequacy and variability across products, it is important to recognize that differences between products may reflect not only intentional formulation targets but also variation in manufacturer resources, production scale, and the reliability of ingredient databases. Companies with larger market share and established research and development programs may have greater capacity to assess nutrient bioavailability and maintain tighter formulation control. In contrast, selective-market manufacturers may rely more heavily on formulation software and theoretical ingredient databases without direct verification of nutrient content, contributing to variability in measured nutrient profiles.

Because many pet food commercial formulations are based on database nutrient values rather than direct analysis of ingredients there can be discrepancies between predicted and actual nutrient concentrations of diets. This can occur due to differences in ingredient sourcing, processing methods, and the degree of analytical verification. Variability in ingredient bioavailability and mineral form, including organic forms (e.g., proteinates) and inorganic forms (e.g., sulfates or oxides), may further influence nutrient bioavailability and complicate the interpretation of laboratory-based analyses. Inconsistencies make it challenging for veterinarians to predict nutrient content based solely on labeling or marketing terms. Therefore, while product labels offer a useful starting point for evaluating diet intent and formulation claims, they should be interpreted in combination with laboratory analyses or manufacturer-supplied nutrient data whenever available especially when considering recommendations for senior dogs with changing nutritional needs.

In conclusion, senior diets should not be recommended based on age alone but rather on nutrient profiles relevant to the individual patient as there are no defined parameters for senior diets. Clinically, veterinarians should assess the nutrient composition of each product to ensure it aligns with the needs of aging dogs. Goals for managing senior dogs include maintaining lean body mass, preventing obesity, and supporting organ and immune function. While many senior dogs may achieve these goals when fed a diet that provides adequate, high-quality protein; moderate fat with omega-3 fatty acids; and sufficient fiber to promote digestive health, the “senior” label alone does not guarantee these characteristics. Given the variability observed, collaboration between veterinarians and pet owners to select diets from manufacturers with strong quality control and transparent nutrient data remains essential. This study demonstrates a lack of consensus among dog food companies regarding nutrient modifications in senior diets, making it difficult for veterinarians and pet owners to anticipate the nutritional profile of a product marketed for senior dogs. These findings emphasize the importance of rigorous quality-control practices in ensuring that diets meet the nutrient requirements established by AAFCO.

## 6 Limitations

This study has several limitations that should be considered when interpreting the results. Diets were obtained from two pet food stores in Fort Collins, CO. Although most brands represented were nationally available, the inclusion of several locally distributed brands may limit generalizability to areas where these products are not sold. Because only a single lot of each product was analyzed, batch to batch variability was not assessed. The nutrient analysis did not include all nutrients listed under AAFCO requirements, so there may be additional differences beyond our findings. Specifically, amino acids, individual essential fatty acids, chloride, iodine, choline, and vitamins were not measured. These nutrients play important roles in supporting muscle maintenance, immune function, and metabolic health in senior dogs, and their inclusion in future analyses would provide a more comprehensive understanding of diet adequacy. Only crude fiber was measured whereas measuring total dietary fiber would have provided a more complete representation of the dietary fiber content.

Detailed ingredient characterization such as primary protein source, ingredient quality, and the use of organic vs. inorganic mineral supplements was not included in this analysis. The study focused on measured nutrient composition rather than formulation inputs, and complete ingredient information was not available for all products. The original ingredient file from 2023 was lost, and although we contacted all manufacturers represented in the dataset, few responded and even fewer were able to provide archived ingredient lists from that year. Furthermore, ingredient lists alone cannot reliably indicate nutrient concentration, digestibility, or bioavailability, and “ingredient quality” lacks an objective definition without targeted testing. For these reasons, ingredient-level comparisons were not undertaken, and the analysis was restricted to laboratory-measured nutrient values.

The number of parameters analyzed and the range of product lines and manufacturers included were also limited. Diets were selected to represent a cross-section of adult and senior products available to consumers rather than an exhaustive sample. Due to the randomization process focusing on marketing parameters (adult or senior), there was an uneven representation of dry and canned diets. These imbalances, along with the relatively small number of companies represented, limit the statistical power to detect some differences and could have influenced the observed nutrient ranges.

Market-segment classification represents another limitation of this study. Because not all pet food companies publicly report revenue, we applied secondary criteria, including retail distribution to categorize brands when revenue data were unavailable. These categories therefore represent broad approximations of market positioning rather than precise economic distinctions. Market segmentation in the pet food industry is complex, and brands may operate across multiple channels or shift distribution strategies over time; thus, these classifications may not fully capture the nuances of company size or market influence.

Interpretation of AAFCO non-compliance also requires caution. As a descriptive, exploratory study, no conclusions can be drawn regarding the cause of non-compliance in diets that did not meet AAFCO nutrient requirements. The study was not designed to determine the reasons for non-compliance; therefore, these findings should not be interpreted as evidence of formulation issues, ingredient variability, or manufacturing errors, which would require targeted evaluation of production processes and multiple product lots.

Finally, clinical outcomes associated with feeding these diets were not evaluated, so conclusions are limited to nutrient composition alone. Adult and senior diets were not matched pairs of identical formulations differing only by marketed age group, which may have influenced the comparisons. Only seven companies of the 25 brands in our dataset had both an adult and a senior product. The results may be confounding, as the observed differences could reflect company specific practices rather than broader trends between adult and senior diets. The analyses were intended to be exploratory and descriptive, and results should be interpreted as hypothesis generating rather than confirmatory. Future studies should incorporate broader sampling across brands, expanded nutrient panels, paired analyses of adult and senior formulations, balanced representation of dry and canned products, and evaluation of clinical outcomes.

## Data Availability

The original contributions presented in the study are included in the article/Supplementary material, further inquiries can be directed to the corresponding author.
