# Supplementary material for: Exploratory analysis of nutrient composition of adult and senior dog diets
Source: Front Vet Sci. 2025 Dec 18;12:1717409. doi: 10.3389/fvets.2025.1717409 (PMC12757753; doi:10.3389/fvets.2025.1717409)
Supplement: Supplementary file 1 [file Table_1.docx]

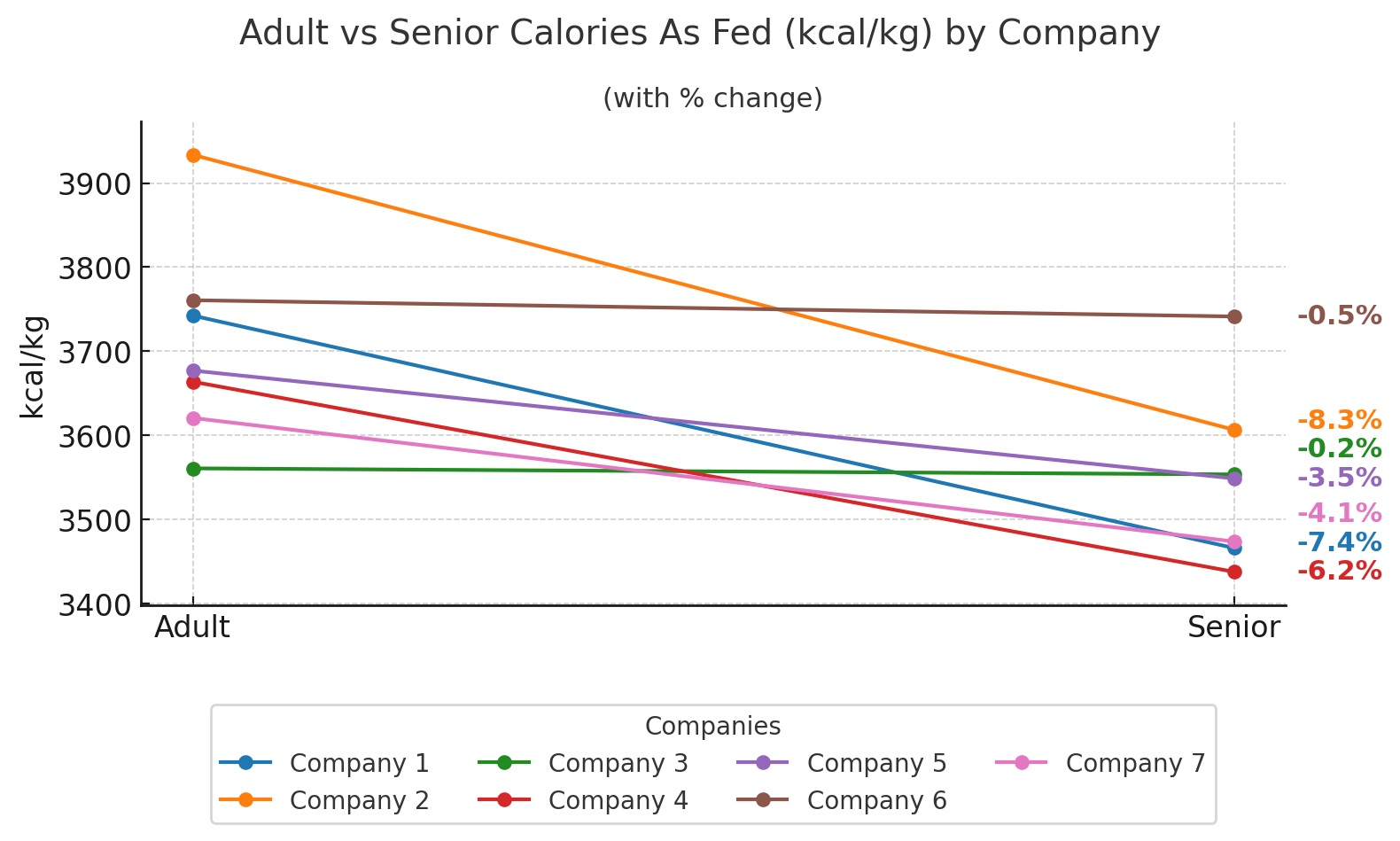


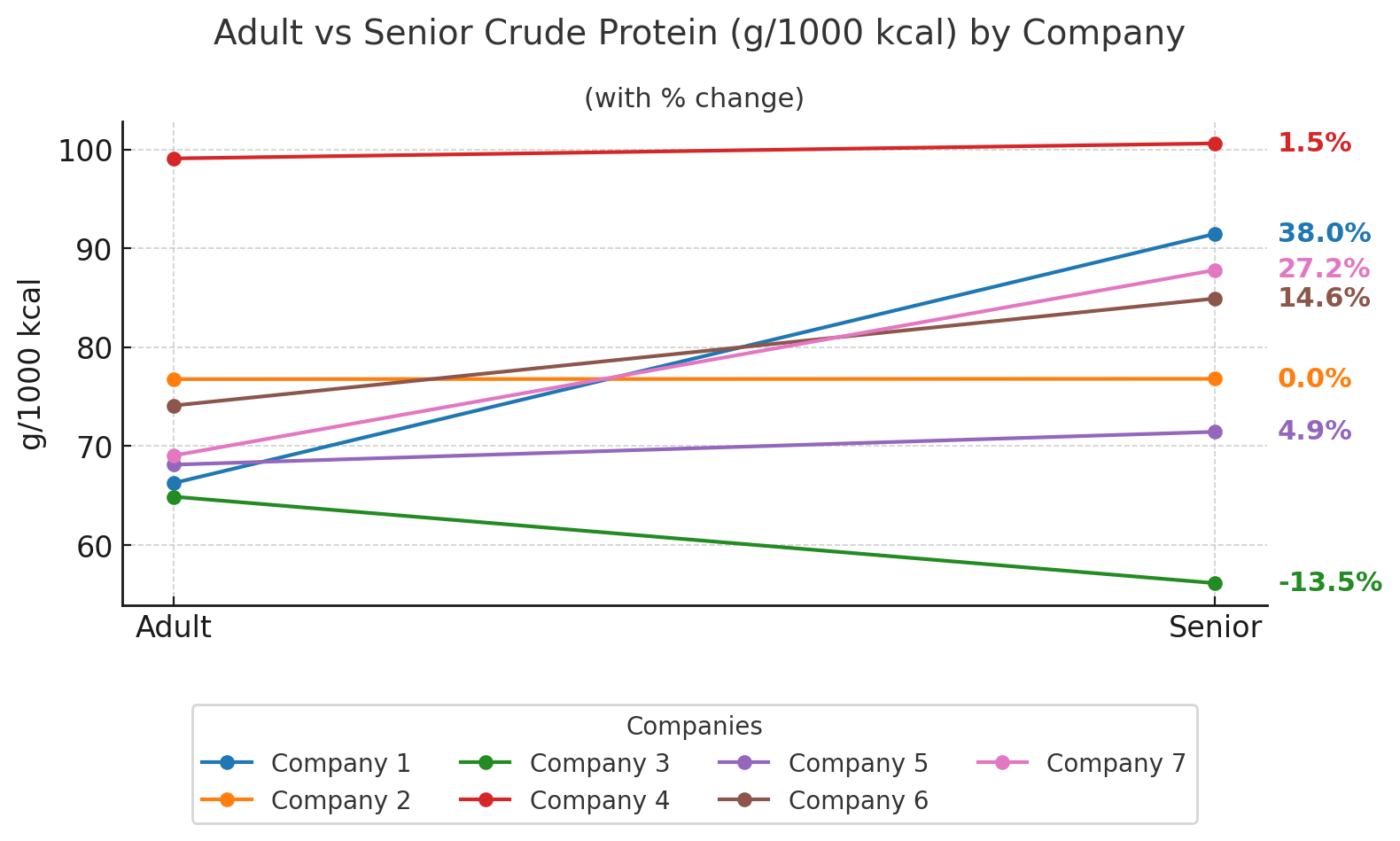


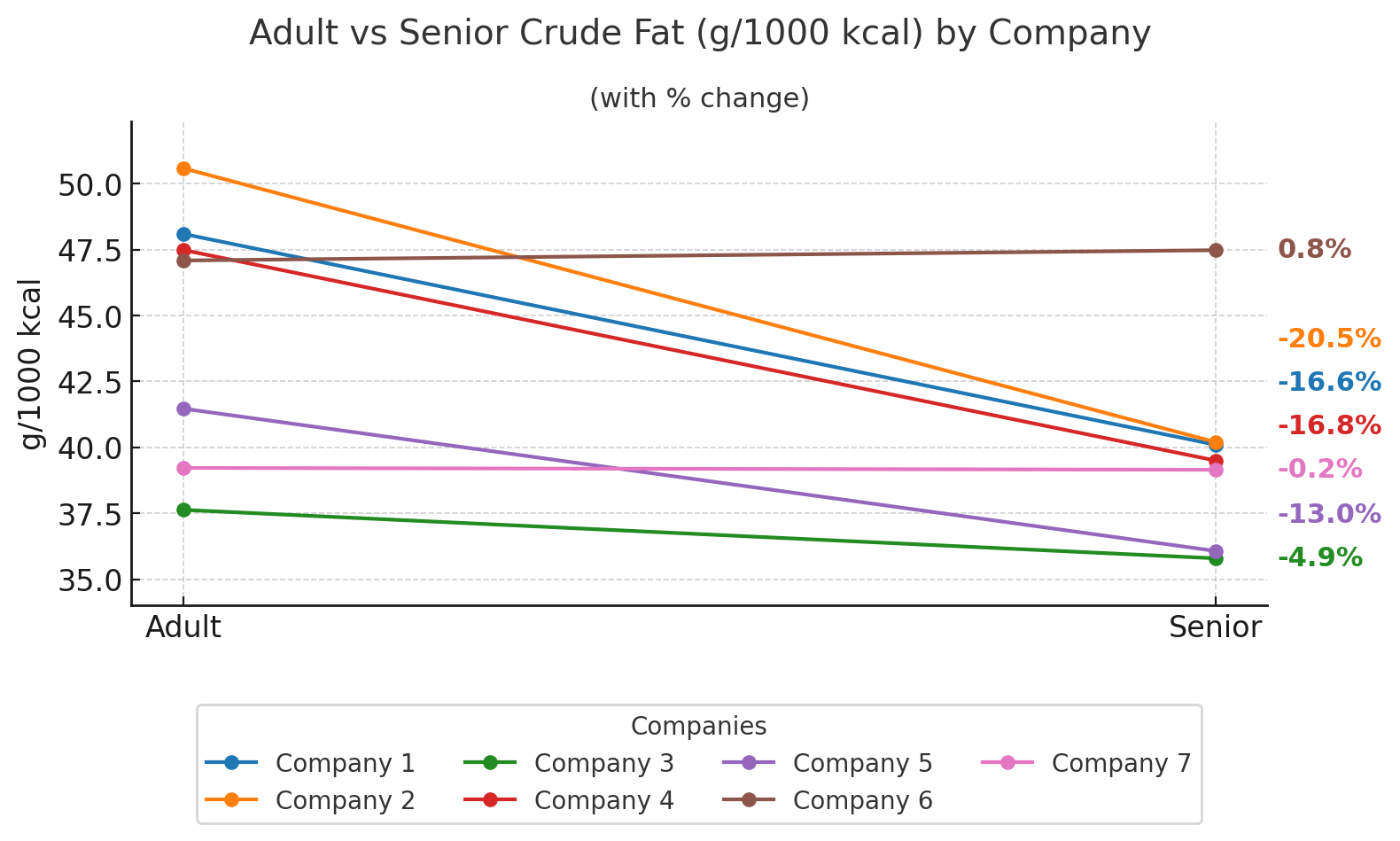


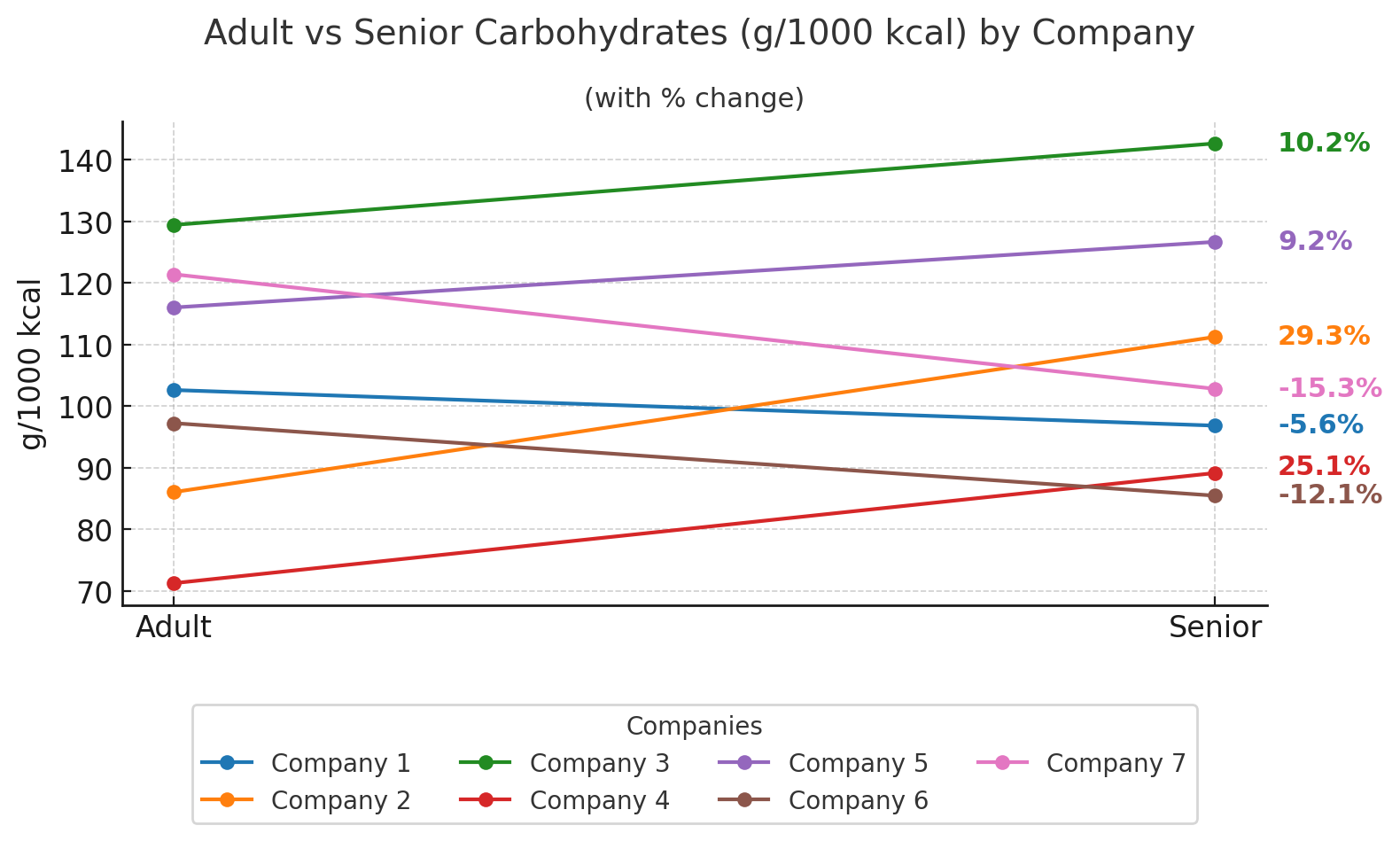


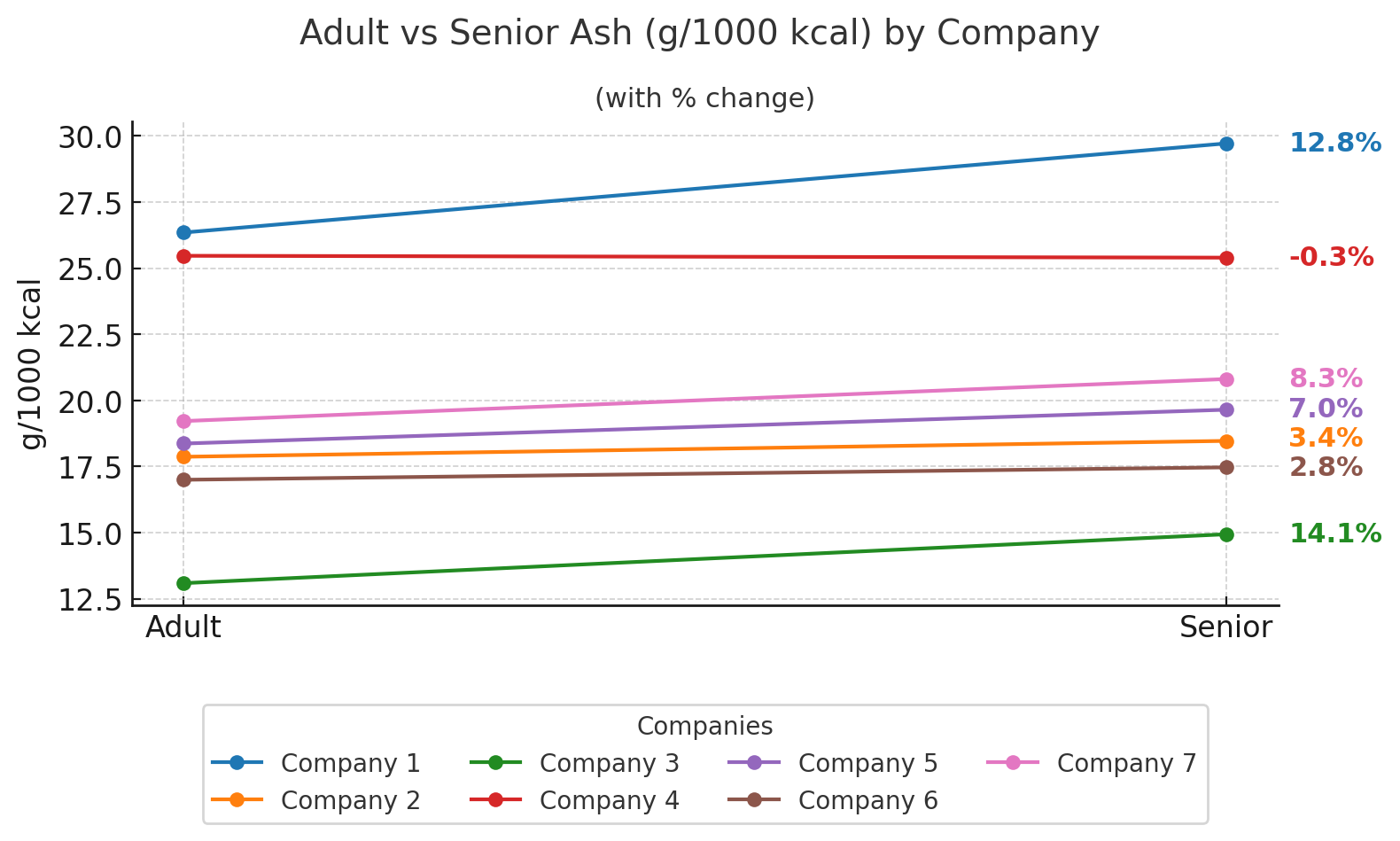


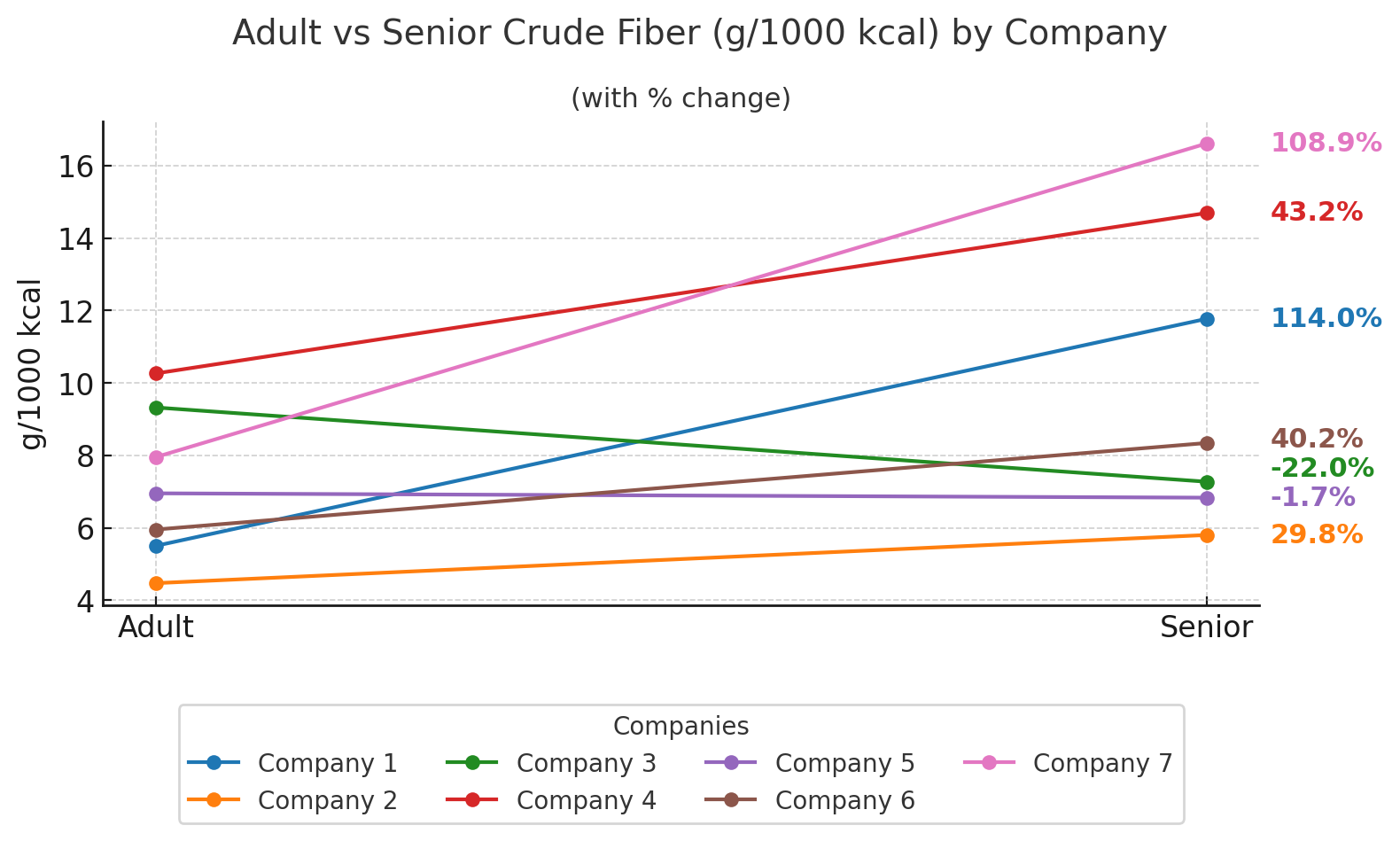


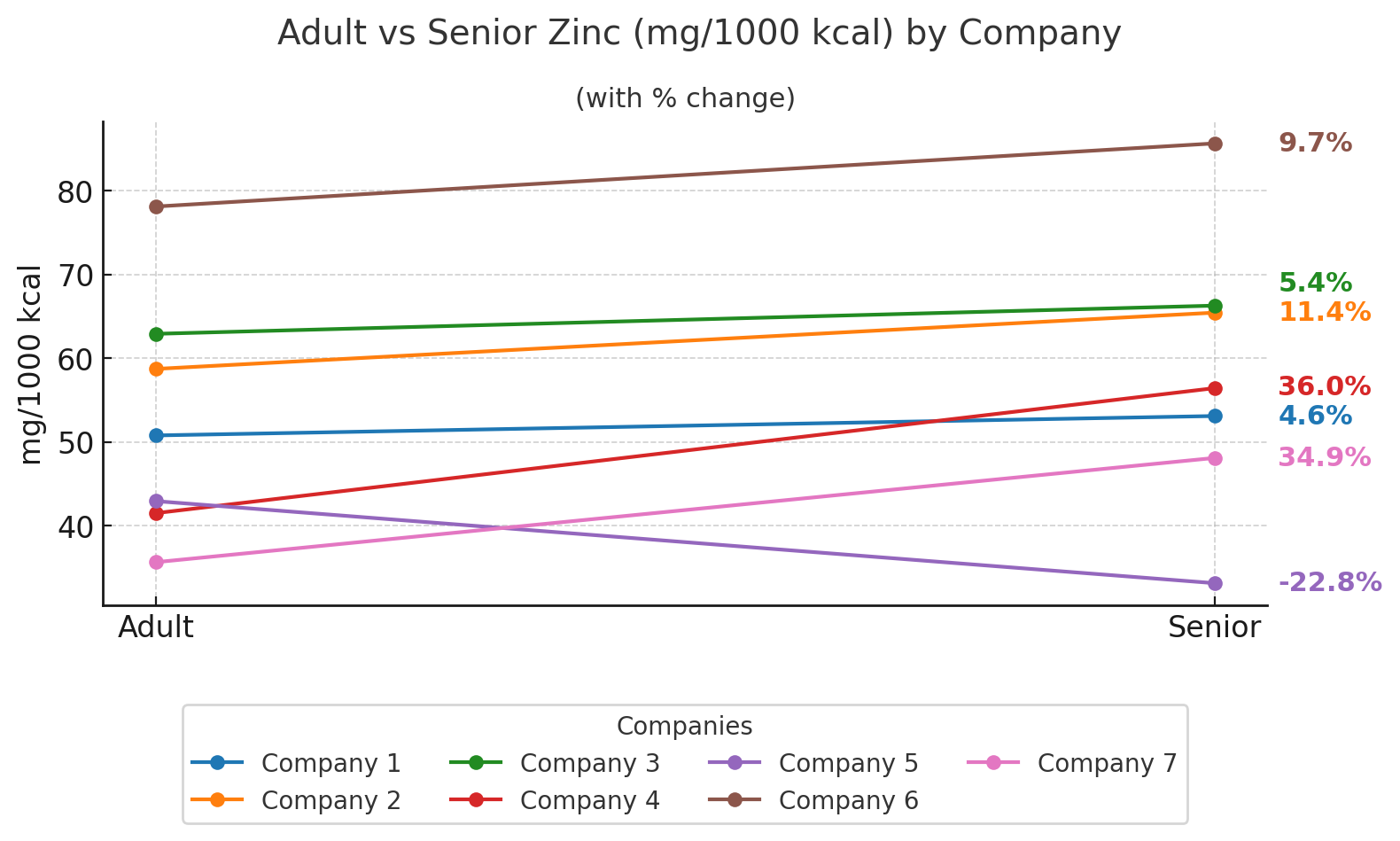


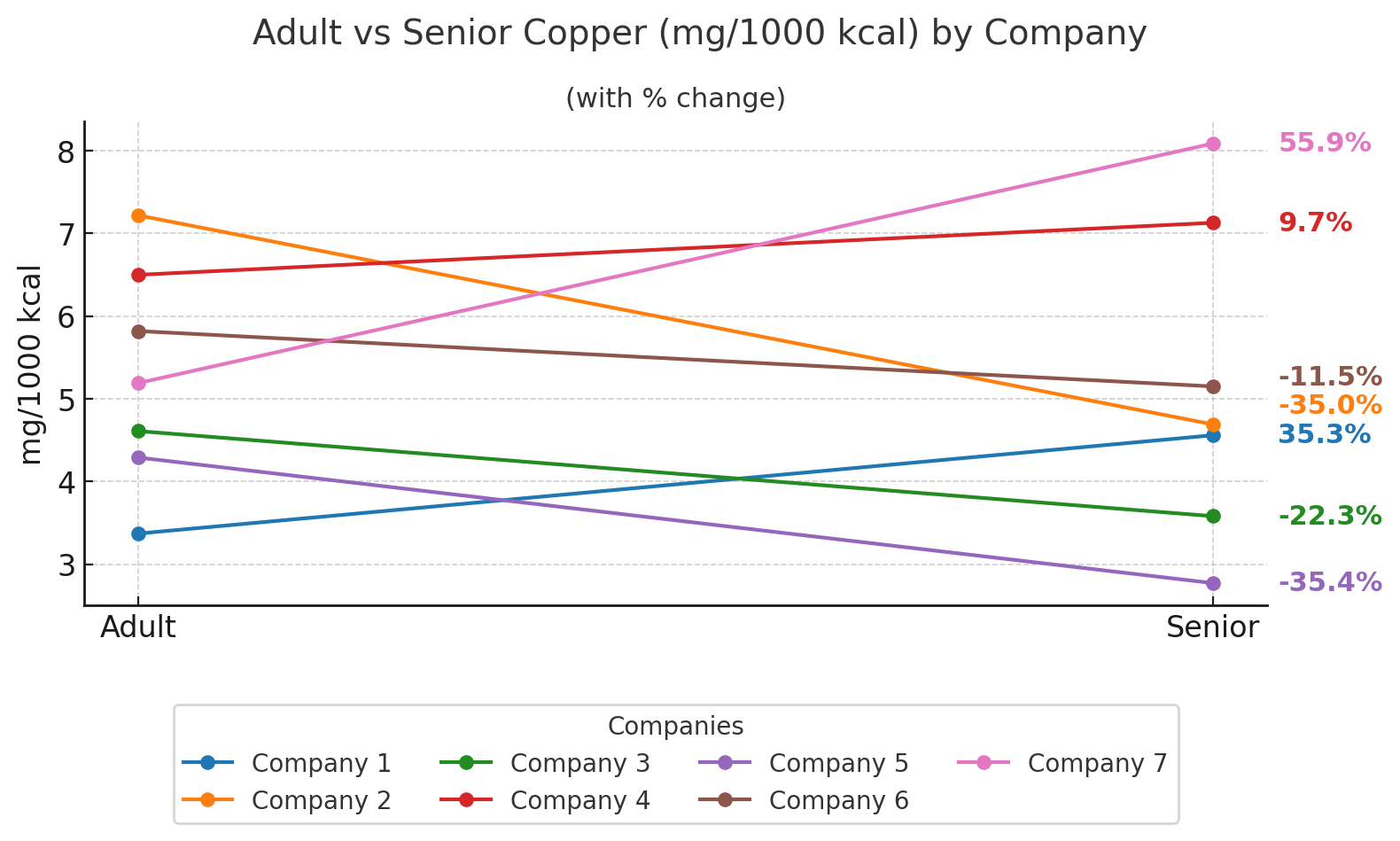


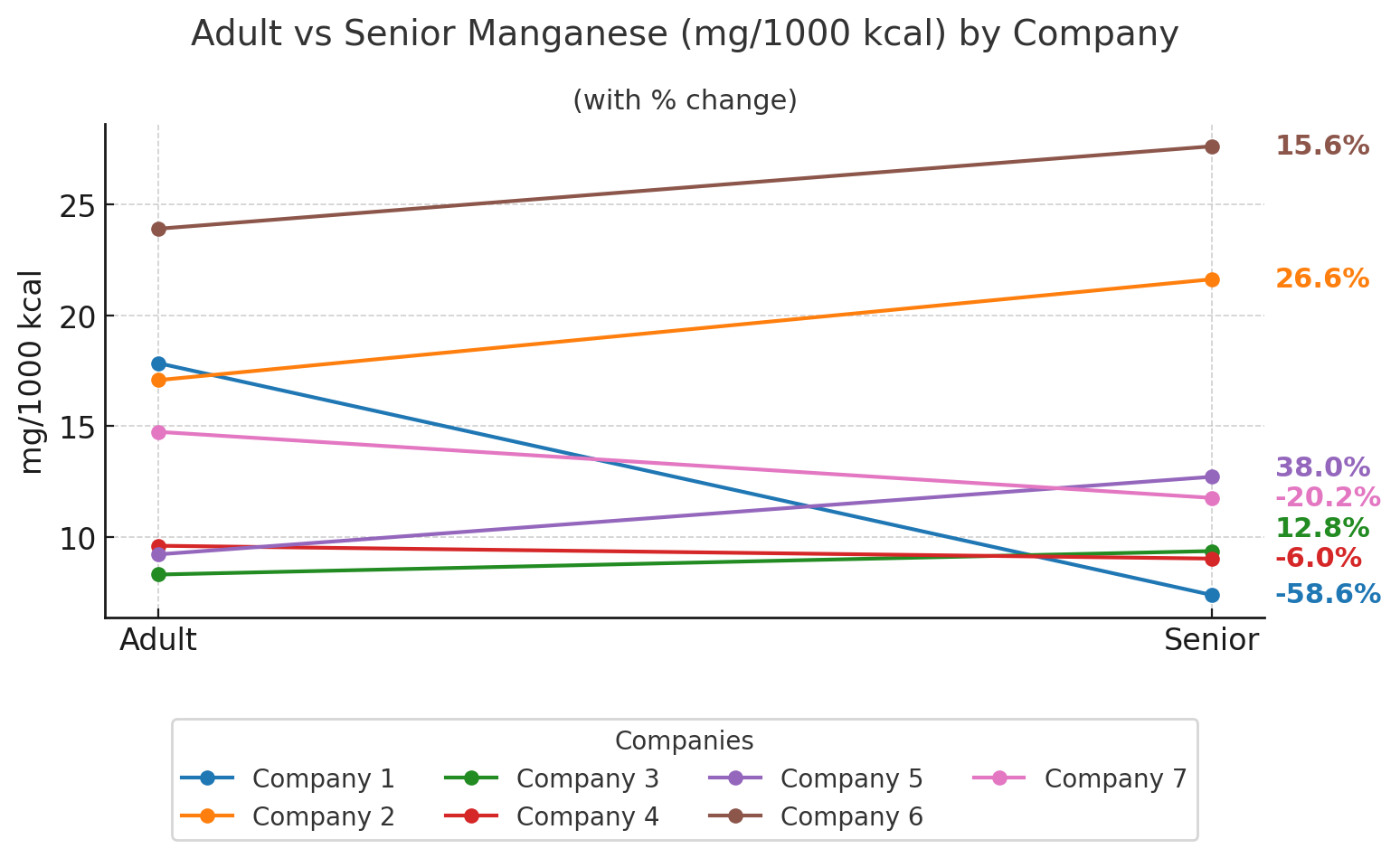


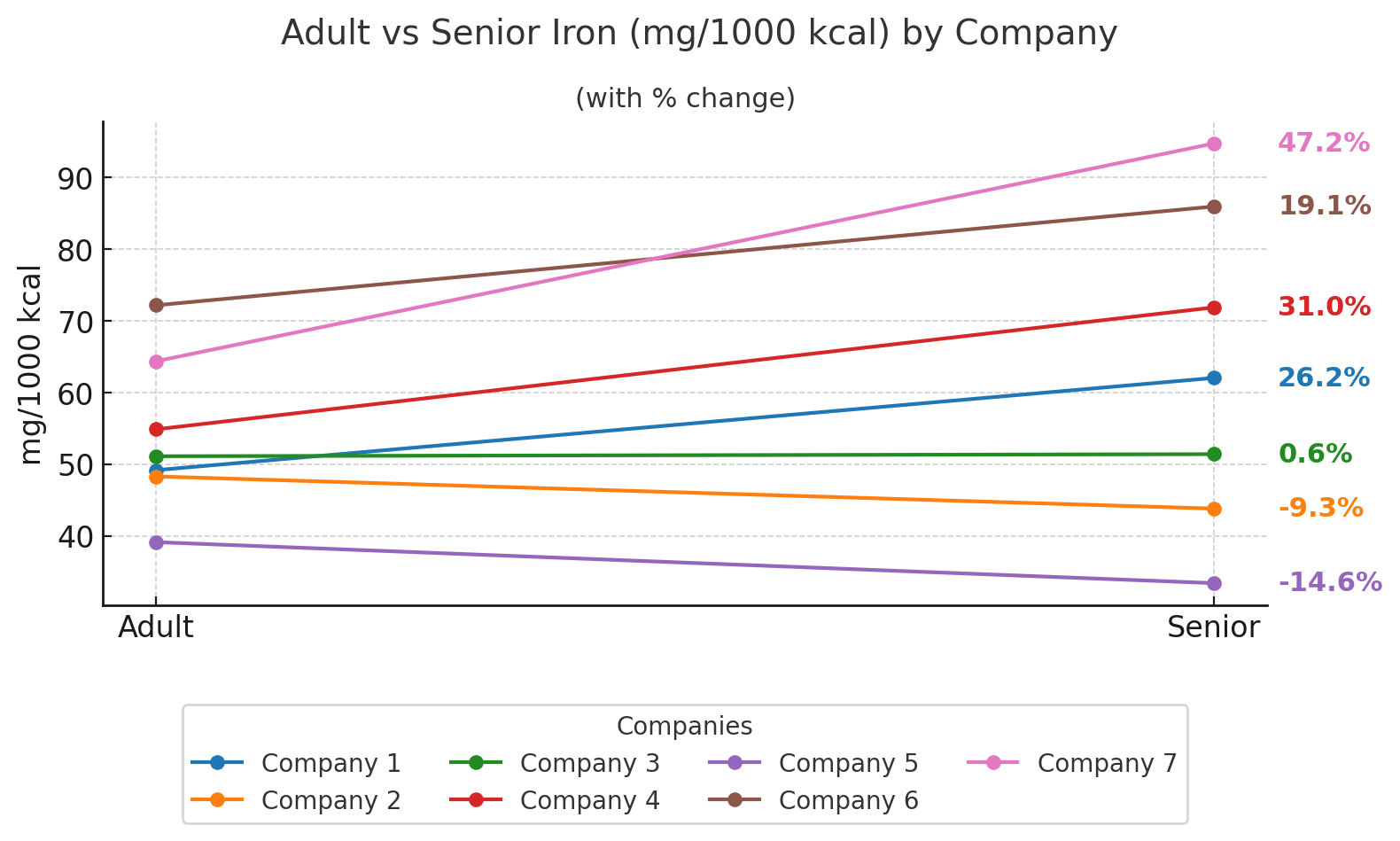


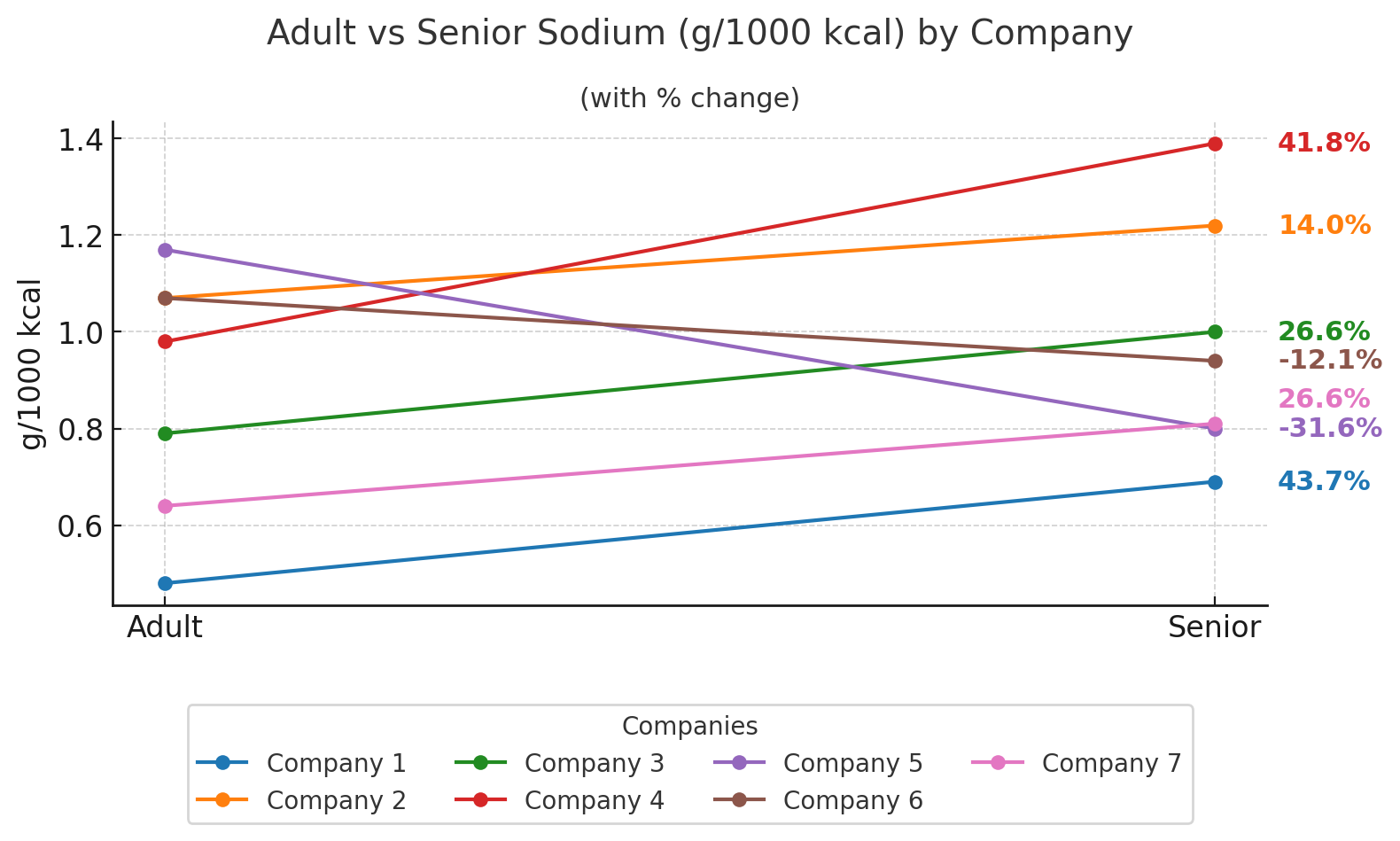


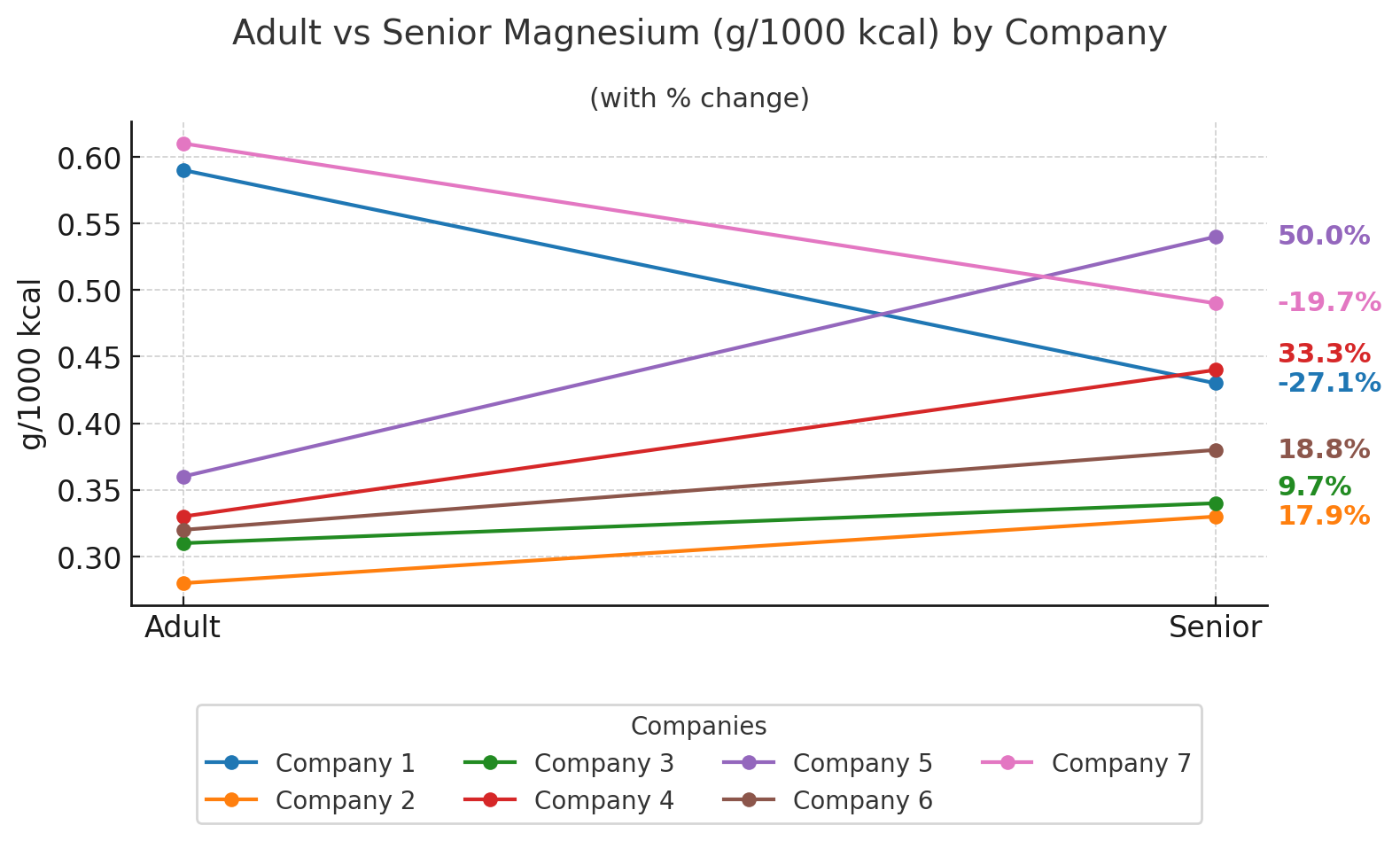


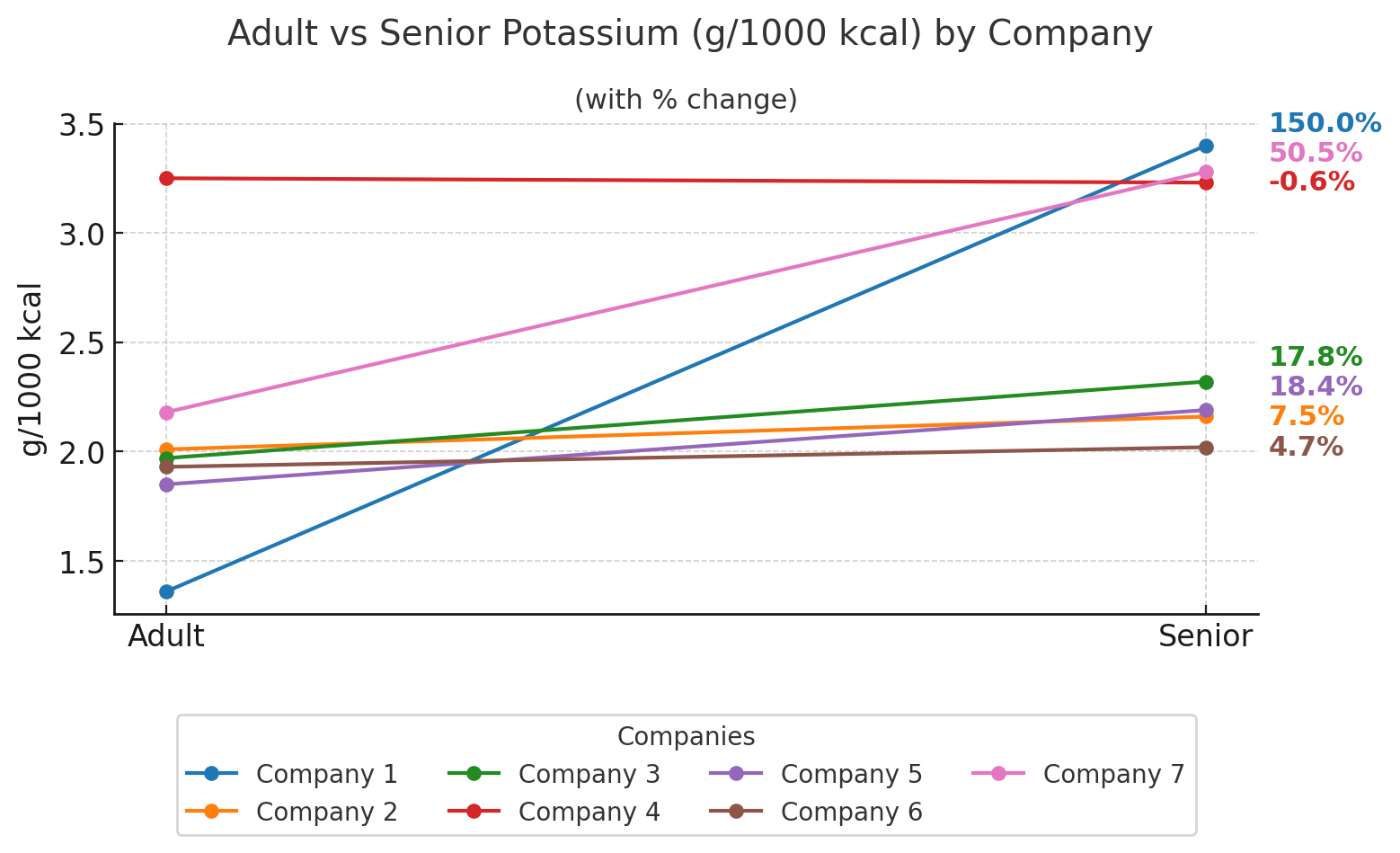


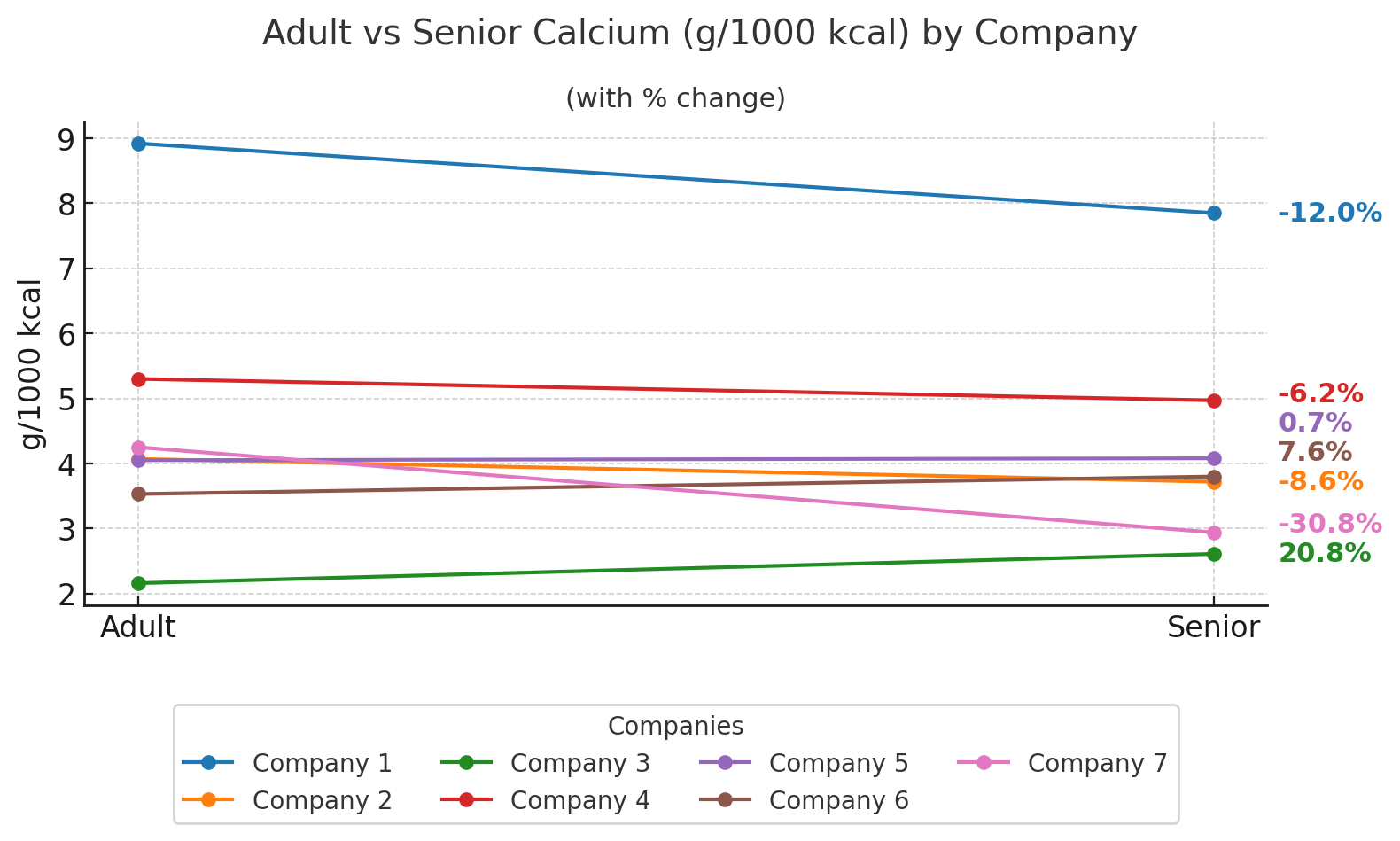


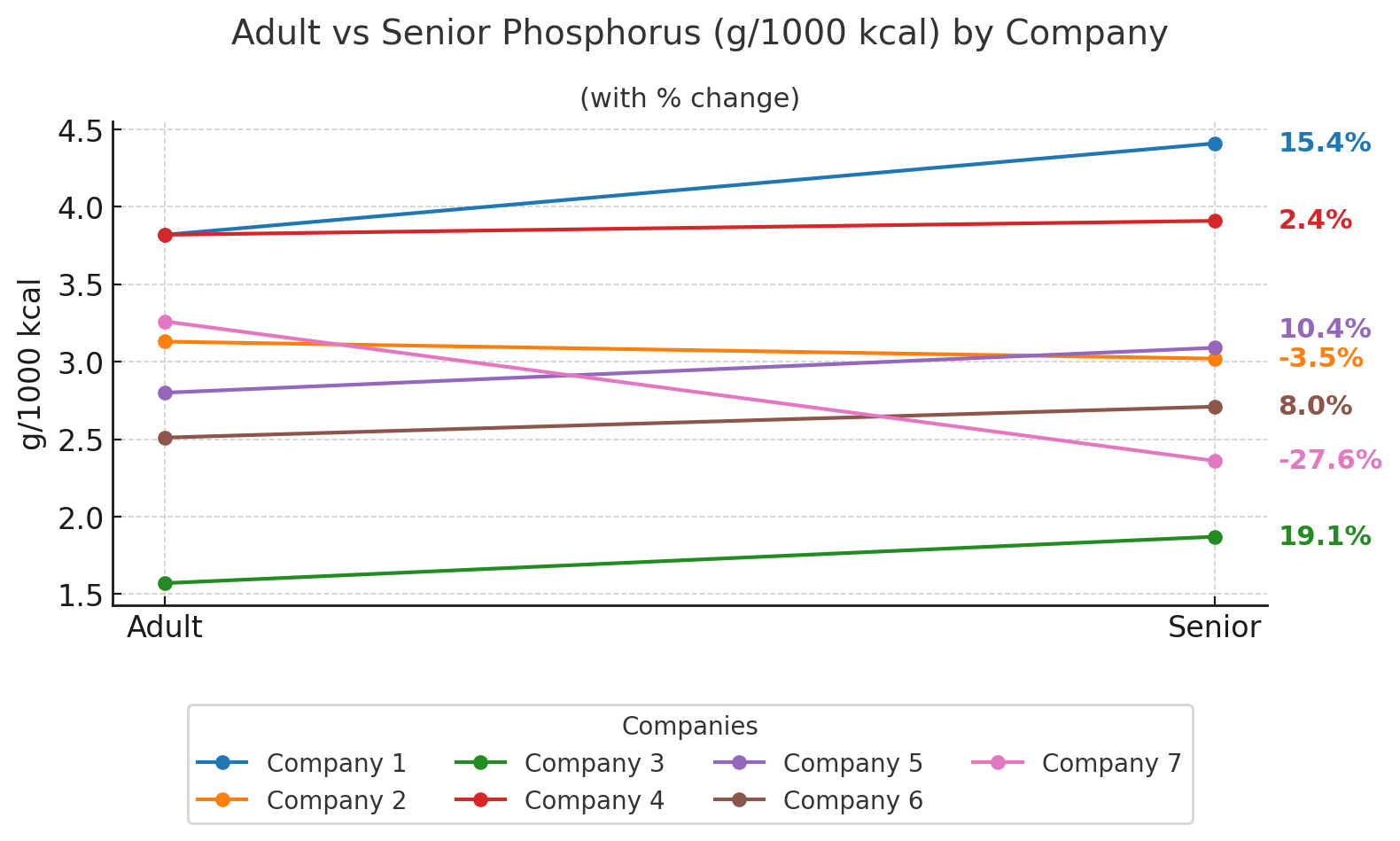


**Supplementary figure 1**: Comparison of nutrient content between adult and senior diets by company. Each colored line represents an individual company’s products, connecting adult and senior formulas for visual comparison. The lines do not represent time but serve as a visual aid to highlight the direction and magnitude of change within each company between their adult and senior diets. Percent change values for each company are shown to the right of the figure.


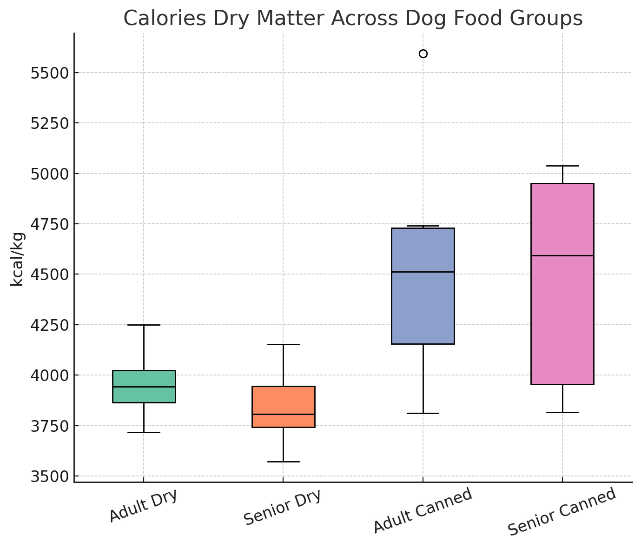

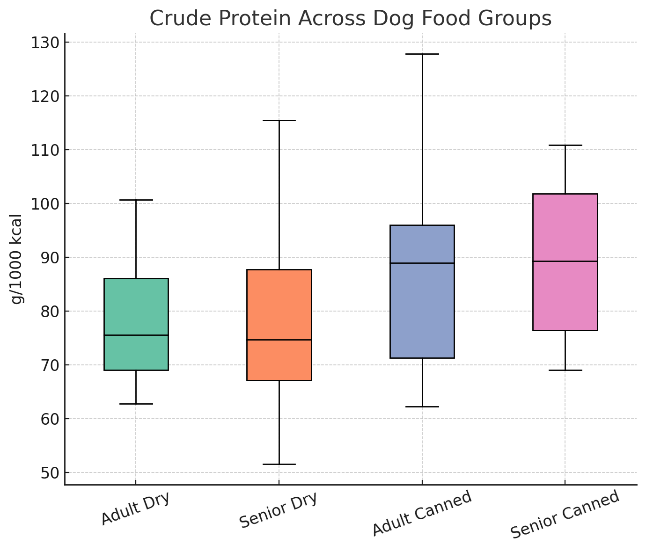


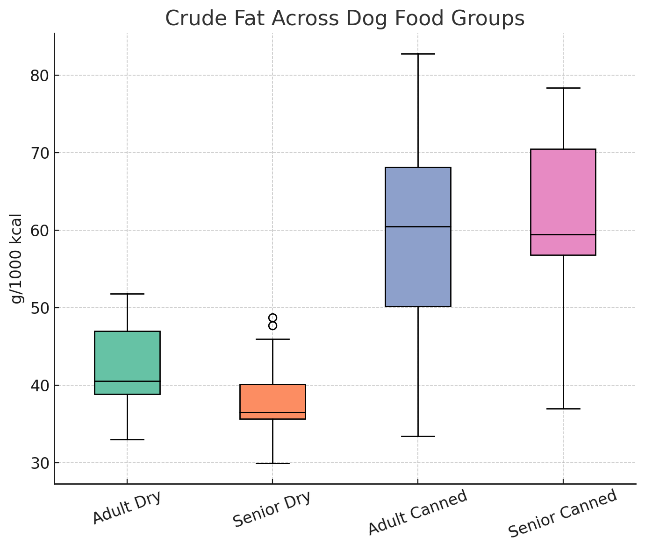

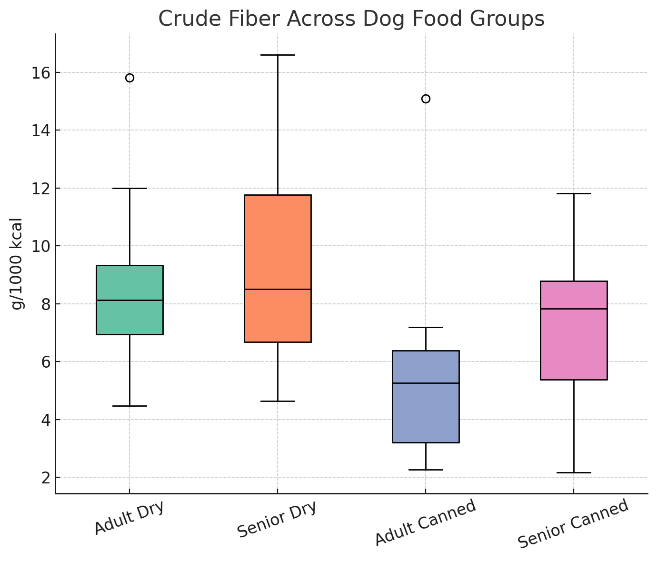


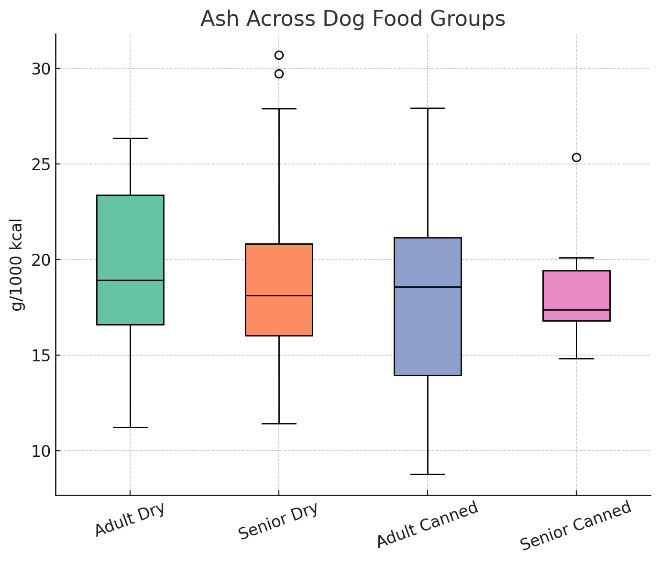

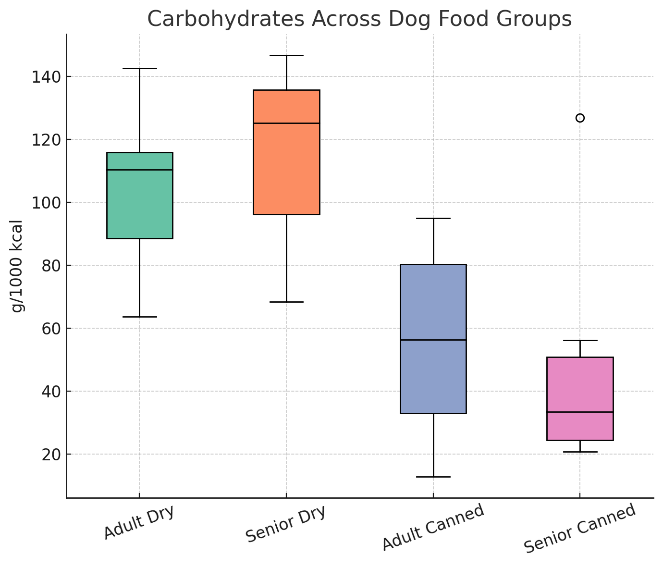


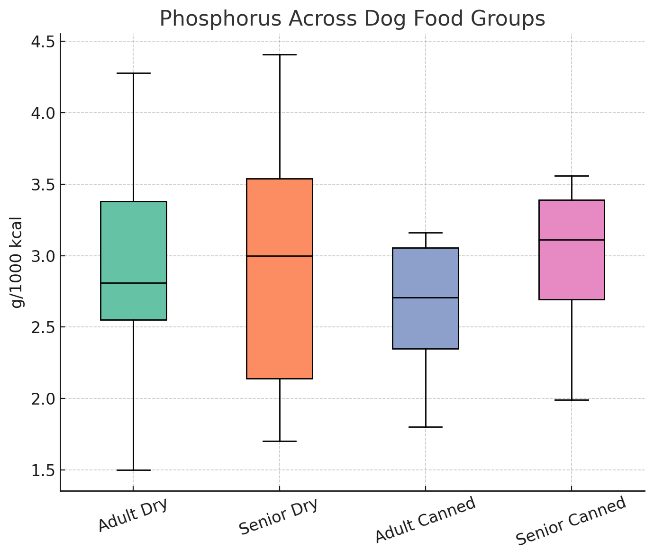

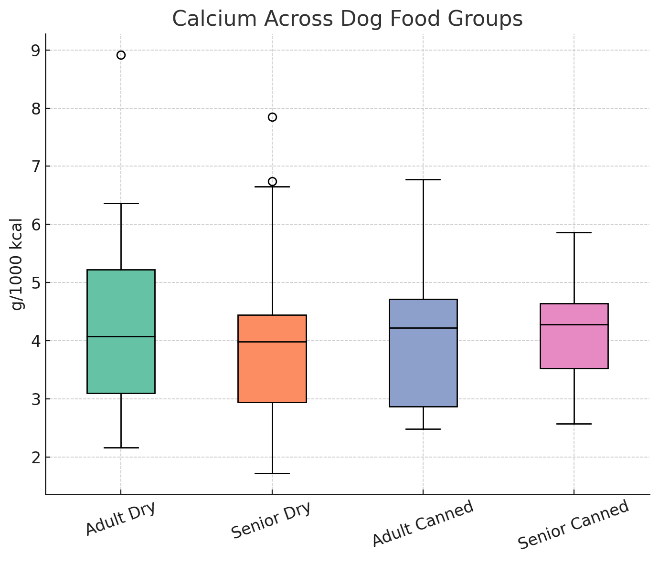


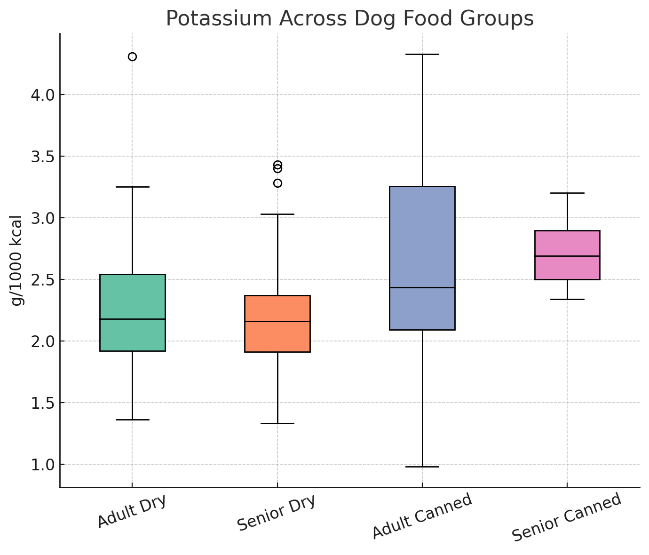

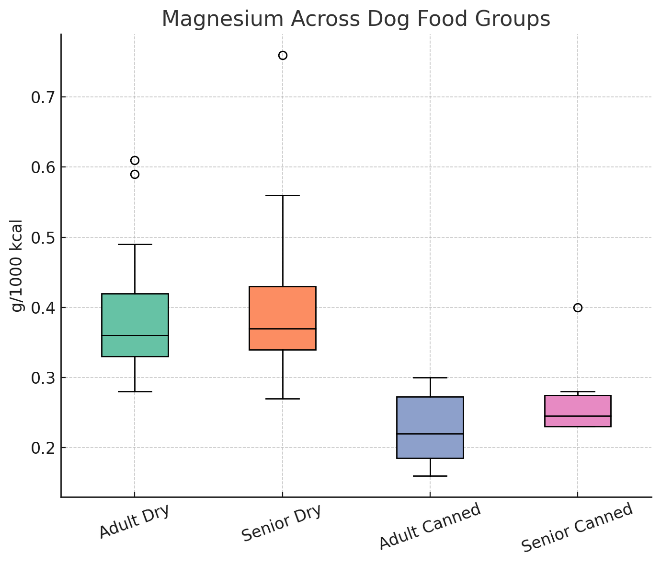


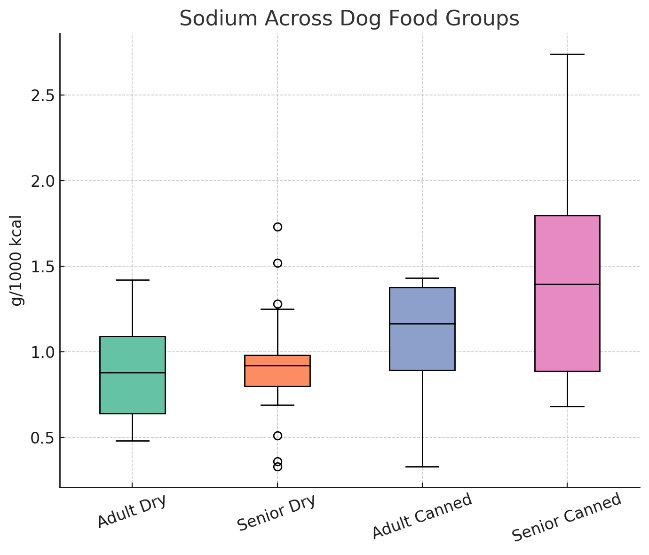

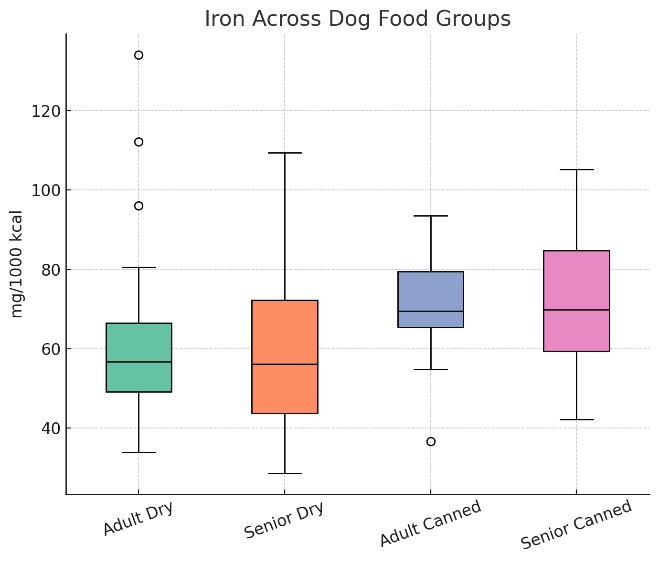


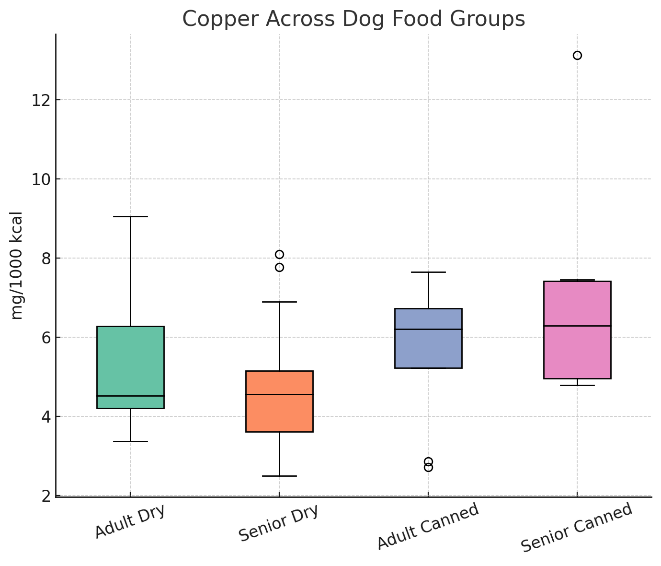

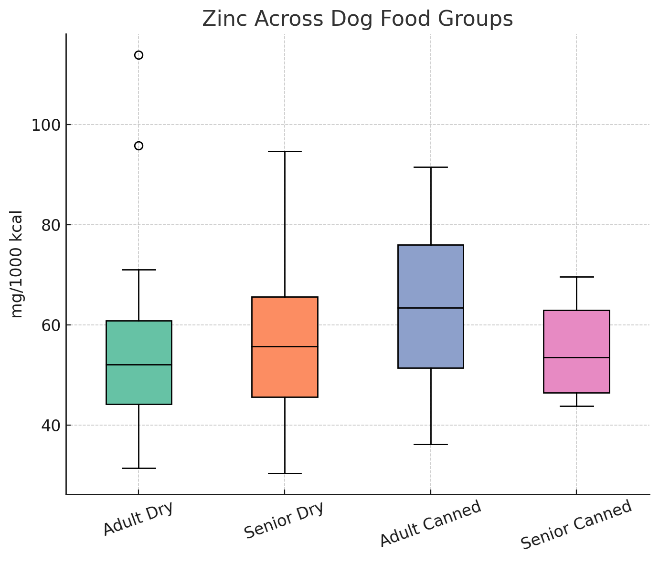


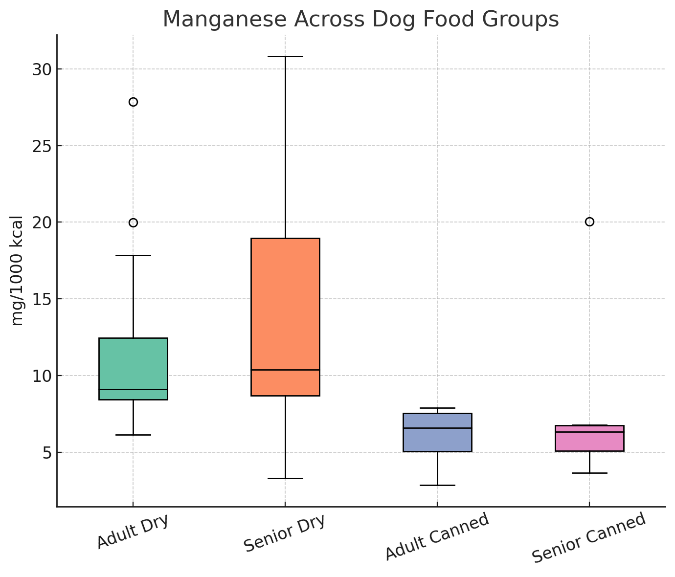


**Supplementary figure 2:** Boxplot graphs comparing all nutrients between adult dry, senior dry, adult canned and senior canned diets. Graphs show distribution of nutrients, median, range and middle 50% quartiles. Outliers are represented as dots outside of the whiskers.
