# Supplementary material for: Exploratory analysis of nutrient composition of adult and senior dog diets
Source: Front Vet Sci. 2025 Dec 18;12:1717409. doi: 10.3389/fvets.2025.1717409 (PMC12757753; doi:10.3389/fvets.2025.1717409)
Supplement: Supplementary file 2 [file Table_2.docx]

|  | Nutrient means | | | | | | | |
| --- | --- | --- | --- | --- | --- | --- | --- | --- |
|  |  |  | Adult | | Senior | |  |  |
| Nutrients | All adult | All senior | Dry | Canned | Dry | Canned | All dry | All canned |
| CP g/1000kcal | 80.9 | 79.9 | 78.2 | 88 | 77.6 | 89.4 | 77.9 | 88.6 |
| Fat g/1000kcal | 46.8 | 42.2 | 42.4 | 58.2 | 37.8 | 60.7 | 39.9 | 59.3 |
| CF g/1000kcal | 7.6 | 9.1 | 8.2 | 5.9 | 9.5 | 7.2 | 8.9 | 6.5 |
| Ash g/1000kcal | 19.1 | 19.2 | 19.4 | 18.1 | 19.3 | 18.6 | 19.4 | 18.3 |
| Carb g/1000kcal | 91.1 | 103.3 | 104.4 | 56.3 | 116.4 | 48.8 | 110.9 | 53.1 |
| P g/1000kcal | 2.8 | 2.9 | 2.9 | 2.6 | 2.9 | 3 | 2.9 | 2.8 |
| K g/1000kcal | 2.4 | 2.4 | 2.3 | 2.6 | 2.3 | 2.7 | 2.3 | 2.7 |
| Mg g/1000kcal | 0.3 | 0.4 | 0.4 | 0.2 | 0.4 | 0.3 | 0.4 | 0.2 |
| Ca g/1000kcal | 4.2 | 4.1 | 4.3 | 4.1 | 4.1 | 4.2 | 4.2 | 4.1 |
| Na g/1000kcal | 0.9 | 1 | 0.9 | 1.1 | 0.9 | 1.5 | 0.9 | 1.2 |
| Fe mg/1000kcal | 65.4 | 62.8 | 64 | 69.1 | 60.5 | 72.1 | 62.1 | 70.4 |
| Mn mg/1000kcal | 10 | 12.4 | 11.5 | 6.1 | 13.4 | 8 | 12.5 | 6.9 |
| Cu mg/1000kcal | 5.3 | 5.1 | 5.2 | 5.7 | 4.7 | 7.1 | 4.9 | 6.3 |
| Zn mg/1000kcal | 58 | 56.5 | 55.8 | 63.7 | 56.8 | 55.1 | 56.4 | 60 |

**Supplementary Table 3**: Means of each analyzed nutrient on a per 1000 kcal basis for each diet category.

|  | Nutrient ranges | | | | | | | |
| --- | --- | --- | --- | --- | --- | --- | --- | --- |
|  |  |  | Adult | | Senior | |  |  |
| Nutrients | All adult | All senior | Dry | Canned | Dry | Canned | All dry | All canned |
| CP g/1000kcal | 62.2 - 127.9 | 51.6 - 115.5 | 62.8 - 100.8 | 62.2 - 127.9 | 51.6 - 115.5 | 69.0 - 110.9 | 51.6 - 115.5 | 62.2 - 127.9 |
| Fat g/1000kcal | 33.0 - 82.8 | 29.9 - 78.4 | 33.0 - 51.8 | 33.4 - 82.8 | 29.9 - 48.7 | 37.0 - 78.4 | 29.9 - 51.8 | 33.4 - 82.8 |
| CF g/1000kcal | 2.3 - 15.8 | 2.2 - 16.6 | 4.5 - 15.8 | 2.3 - 15.1 | 4.6 - 16.6 | 2.2 - 11.8 | 4.5 - 16.6 | 2.2 - 15.1 |
| Ash g/1000kcal | 8.8 - 27.9 | 11.4 - 30.7 | 11.2 - 26.4 | 8.8 - 27.9 | 11.4 - 30.7 | 14.8 - 25.4 | 11.2 - 30.7 | 8.8 - 27.9 |
| Carb g/1000kcal | 12.9 - 142.7 | 20.8 - 146.9 | 63.7 - 142.7 | 12.9 - 95.0 | 68.4 - 146.9 | 20.8 - 126.9 | 63.7 - 146.9 | 12.9 - 126.9 |
| P g/1000kcal | 1.5 - 4.3 | 1.7 - 4.4 | 1.5 - 4.3 | 1.8 - 3.2 | 1.7 - 4.4 | 2.0 - 3.6 | 1.5 - 4.4 | 1.8 - 3.6 |
| K g/1000kcal | 1.0 - 4.3 | 1.3 - 3.4 | 1.4 - 4.3 | 1.0 - 4.3 | 1.3 - 3.4 | 2.3 - 3.2 | 1.3 - 4.3 | 1.0 - 4.3 |
| Mg g/1000kcal | 0.2 - 0.6 | 0.2 - 0.8 | 0.3 - 0.6 | 0.2 - 0.3 | 0.3 - 0.8 | 0.2 - 0.4 | 0.3 - 0.8 | 0.2 - 0.4 |
| Ca g/1000kcal | 2.2 - 8.9 | 1.7 - 7.8 | 2.2 - 8.9 | 2.5 - 6.8 | 1.7 - 7.8 | 2.6 - 5.9 | 1.7 - 8.9 | 2.5 - 6.8 |
| Na g/1000kcal | 0.3 - 1.4 | 0.3 - 2.7 | 0.5 - 1.4 | 0.3 - 1.4 | 0.3 - 1.7 | 0.7 - 2.7 | 0.3 - 1.7 | 0.3 - 2.7 |
| Fe mg/1000kcal | 33.9 - 134.2 | 28.6 - 109.4 | 33.9 - 134.2 | 36.7 - 93.5 | 28.6 - 109.4 | 42.2 - 105.2 | 28.6 - 134.2 | 36.7 - 105.2 |
| Mn mg/1000kcal | 2.9 - 27.9 | 3.3 - 30.8 | 6.1 - 27.9 | 2.9 - 7.9 | 3.3 - 30.8 | 3.6 - 20.1 | 3.3 - 30.8 | 2.9 - 20.1 |
| Cu mg/1000kcal | 2.7 - 9.1 | 2.5 - 13.1 | 3.4 - 9.1 | 2.7 - 7.7 | 2.5 - 8.1 | 4.8 - 13.1 | 2.5 - 9.1 | 2.7 - 13.1 |
| Zn mg/1000kcal | 31.4 - 114.0 | 30.4 - 94.7 | 31.4 - 114.0 | 36.1 - 91.6 | 30.4 - 94.7 | 43.8 - 69.6 | 30.4 - 114.0 | 36.1 - 91.6 |

**Supplementary Table 4**: Analyzed nutrient ranges for each diet category. Results provided on a per 1000 kcal basis.
